# Structure, stability, absorption spectra and aromaticity of the singly and doubly silicon doped aluminum clusters Al_*n*_Si_*m*_^0/+^ with *n* = 3–16 and *m* = 1, 2†

**DOI:** 10.1039/c9ra04004h

**Published:** 2019-08-30

**Authors:** Nguyen Minh Tam, Long Van Duong, Ngo Tuan Cuong, Minh Tho Nguyen

**Affiliations:** Computational Chemistry Research Group, Ton Duc Thang University Ho Chi Minh City Vietnam nguyenminhtam@tdtu.edu.vn; Faculty of Applied Sciences, Ton Duc Thang University Ho Chi Minh City Vietnam; Institute for Computational Science and Technology (ICST) Quang Trung Software City Ho Chi Minh City Vietnam; Faculty of Chemistry and Center for Computational Science, Hanoi National University of Education Hanoi Vietnam; Department of Chemistry, KU Leuven Celestijnenlaan 200F B-3001 Leuven Belgium

## Abstract

Structures of the binary Al_*n*_Si_*m*_ clusters in both neutral and cationic states were investigated using DFT and TD-DFT (B3LYP/6-311+G(d)) and (U)CCSD(T)/cc-pvTZ calculations. Silicon-doped aluminum clusters are characterized by low spin ground states. For small sizes, the Si dopant prefers to be located at vertices having many edges. For larger sizes, the Si atom prefers to be endohedrally doped inside an Al_*n*_ cage. Relative stability, adiabatic ionization energy and dissociation energies of each cluster size were evaluated. A characteristic of most Si doped Al clusters is the energetic degeneracy of two lowest-lying isomers. Calculated results confirm the high stability of the sizes Al_4_Si_2_, Al_12_Si and Al_11_Si_2_^+^ as “magic” clusters, that exhibit 20 or 40 shell electrons and are thermodynamically more stable as compared to their neighbors. Electronic absorption spectra of isoelectronic magic clusters Al_13_^−^, Al_12_Si, and Al_11_Si_2_^+^ that have two pronounced bands corresponding to blue and violet lights, have been rationalized by using the electron shell model. The magnetically included ring current density (MICD) analyses suggest that they are also aromatic structures as a result of the “magic” 40 shell electrons.

## Abstract 2

The isoelectronic “magic” clusters with 40 shell electrons have enhanced thermochemical stability.

## Graphical abstract

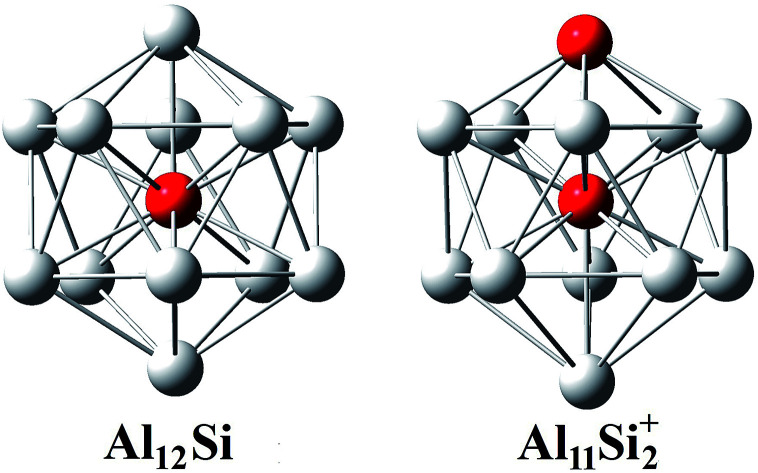

## 1. Introduction

**Scheme 1:**
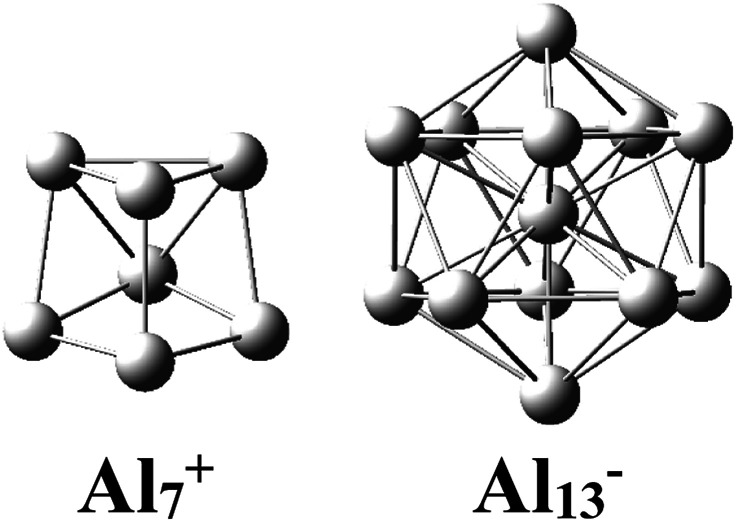
Structural motifs of the pure magic clusters Al_7_^+^ and Al_13_^−^.

There has been considerable interest in the aluminum clusters as witnessed by a large number of experimental and theoretical studies reported on them in the past decades, in part due not only to their appealing physical and chemical properties, but also to their promising abilities for new technological applications.^1–16^ Both the pure cationic Al_7_^+^ and anionic Al_13_^−^ clusters (Scheme 1) were revealed as “magic clusters” with an enhanced thermochemical stability as each possesses a closed electron shell structure including 20 and 40 valence electrons, respectively. The neutral Al_13_, as formed from removal of one electron from the anion Al_13_^−^, was shown to be a superhalogen having a very high electron affinity exceeding those of halogen atoms.^8,17^

Along with pure Al clusters, several doped Al clusters have also been investigated. For alkali and alkali-earth dopants, while Rao *et al.*^18^ presented a theoretical study on their geometries and energetics including adsorption energies, ionization potentials, and electron affinities of Al_*n*_Li and Al_*n*_K, Sun and co-workers^19^ examined Al_*n*_Be using the high accuracy method CCSD(T). Wang and coworkers^20,21^ investigated the Al Zintl anion moieties within the mixed sodium as well as magnesium aluminum clusters Na_*m*_Al_*n*_^−/0^ and Mg_*m*_Al_*n*_^−^ using a combination of anion photoelectron spectroscopy and density functional theory (DFT) calculations. Studies on transition metal (TM) doped Al clusters provide us with a considerable amount of results. For copper derivatives Al_*n*_Cu with *n* = 11–14, unlike the alkali atom-doped counterparts in which the alkali dopant prefers an exohedral position, the copper atom tends to reside inside an Al cage.^22^ For platinum doped aluminum clusters, Zhang *et al.* reported a joint photoelectron spectroscopic and theoretical study of the PtAl^−^ and PtAl_2_^−^ anions.^23^ An experimental study on the argon physisorption ability of TM indicated that Al_*n*_TM^+^ clusters attach one argon atom up to a critical cluster size *n*_crit_, with *n*_crit_ = 16 and *n*_crit_ = 19–21 for TM = V, Cr, and TM = Ti, respectively, and undergo a geometrical transition in going from exohedrally to endohedrally doped clusters in which the TM atom is located inside an Al cage.^24^ Experimental results on Al_*n*_Ti were confirmed by subsequent theoretical DFT computations.^25^

More recently, the influence of spin on the properties of TM doped aluminum clusters in a series of low spin clusters was found in which a prominent odd–even oscillation in all calculated properties supports the presence of jellium shell structure. The electron jellium model considers a cluster of atoms as a superatom where the cores of constituent atoms form a constant positive background, the “jellium density”, and only valence electrons are treated explicitly. Under the model, the shell molecular orbitals of the clusters are denoted as 1S, 1P, 1D, 2S, 2P, 1F, … (usually the notation 1s, 1p, 1d, 2s, 2p, 1f, … have also been used), rather than as the atomic orbitals 1s, 2s, 2p, 3s, 3p, 3d, … of hydrogen-like atoms. High spin ground state isomers show more smooth trends similar to the bulk materials.^26^ Geometrical and electronic structures as well as physical and chemical properties of Al clusters doped by various common nonmetal dopants have also abundantly been investigated.^27–45^

Interaction of hydrogen molecules with Al_*n*_Rh_2_^+^ with *n* = 10–13 clusters was recently studied by mass spectrometry and infrared multiple photon dissociation (IRMPD) spectroscopy.^46^ A comparison of the IRMPD spectra with predictions obtained using DFT calculations showed that for *n* = 10 and 11, a single H_2_ molecule dissociates upon binding to the cluster, whereas for *n* = 12 and 13, it adsorbs molecularly. Upon adsorption of a second H_2_ molecule to the cation Al_12_Rh_2_^+^, both hydrogen molecules dissociate. These results point out a peculiar catalytic effect of doped Al clusters.

Silicon, being contiguous to Al in the periodic table and having one valence electron more than Al, has been and still is massively used in the semiconductors and optoelectronic industries. A mixture of aluminum–silicon atoms gives rise to some quite interesting compounds as previous studies indicated some remarkable properties of Al–Si nanomaterials. For instance, interaction between a Si nanowire and one Al-atom was found to increase the electrical conductivity as compared to pristine Si-nanowire.^47^ Al atoms form an ordered array of magic clusters on the surfaces of Si(111)^48^ and a persistent formation of Al–Si nanowires was reported.^49^ However, understanding of geometrical and electronic structures and physico-chemical properties of binary Al–Si clusters remains limited. Earlier studies mainly examined the Al doped Si clusters.^50–52^ whereas only a few studies on the Si doped Al clusters were performed and concentrated on high symmetry structures^53,54^ that are not necessarily the most thermodynamically stable structural motifs.

In view of the scarcity of reliable results on mixed Al–Si clusters, we set out to carry out a systematic investigation, using both density functional theory (DFT) and wavefunction methods, on a series of small singly and doubly silicon doped aluminum cluster Al_*n*_Si_*m*_, with *n* = 3–16 and *m* = 1–2, in both neutral and cationic states. Geometries of the most stable equilibrium structures and their energetic parameters are determined by using both DFT and coupled-cluster theory CCSD(T) methods. It is of importance to emphasize the similarities and differences between the behavior of Al–Si clusters that have a similar size and number of electrons. As for a typical example, as the isoelectronic species Al_13_^−^, Al_12_Si and Al_11_Si_2_^+^ have closed electronic shells in the jellium model (40 valence electrons), we thus model their optical absorption spectra by using the time-dependent DFT (TD-DFT).

## 2. Computational methods

All standard electronic structure calculations are carried out using the GAUSSIAN 09 package.^55^ The search for possible structures of clusters is conducted using different approaches. First, we generate many initial structures of Al_*x*_Si_*y*_, denoted (*x*, *y*) for simplicity, by modifying the known global minima (GM) geometries taken from the literature as follows: (a) addition of a Si at different positions of the (*x*, *y* − 1) GM; (b) addition of Al at different positions of the (*x* − 1, *y*) GM; (c) Al-to-Si substitution in (*x* + 1, *y* − 1) GM, and Si-to-Al substitution in (*x* − 1, *y* + 1) GM. These initial isomers are rapidly optimized using DFT with the hybrid B3LYP functional in conjunction with the 6-31G(d) basis set. The lowest-lying isomers then become the inputs for the following search using a stochastic algorithm, with the aim to survey all possible lower-lying isomers of each Al_*n*_Si_*m*_ size.

Geometries and harmonic vibrational frequencies of the lower-lying isomers having relative energies of <10 eV of Si_*n*_Al_*m*_ at the small sizes (*n* ≤ 4) and their corresponding cations are then reoptimized with the higher spin states (triplet and quintet for closed shell clusters, and quartet and sextet for open shell clusters). However, no high spin states having a remarkable stability are found, due to the fact that both Al and Si are basically non-magnetic atoms. It can thus be predicted that the larger sizes of Al_*n*_Si_*m*_ also exist as non-magnetic clusters, and therefore we can neglect the calculations of their high spin states due to the limitations of our computational resource.

The local minima with relative energies of <5 eV with respect to the corresponding lowest-lying isomer of each size are considered again and reoptimized using the same B3LYP functional but with the larger 6-311+G(d) basis set. Harmonic vibrational frequencies are again calculated at this level in order to identify the obtained structures as local minima on the potential energy surface and to evaluate zero-point energy corrections. To obtain more reliable energetic parameters, the lower-lying isomers of each size having relative energies within 1.0 eV are selected for single point electronic energies using the coupled-cluster theory CCSD(T) with the correlation consistent cc-pVTZ basis set, based on B3LYP optimized geometries. The unrestricted formalism (UHF, UB3LYP, UCCSD) is employed for open-shell structures.

In an attempt to probe further the effects induced by the Si dopants on the electronic structure, we perform time-dependent density functional theory (TD-DFT) calculations using the same functional/basis set and the optimized geometries of the closed-shell isoelectronic Al_13_^−^, Al_12_Si and Al_11_Si_2_^+^ species. These calculations also allow us to construct their absorption spectra. For each species, the number of electronically excited states considered is large enough for the spectra spread out up to a wavelength of 250 nm. The shape of molecular orbitals involved in the electron shells and the density of states of the above clusters are also plotted assisting the analysis of their spectra.

In addition, we use the magnetically included ring current density (MICD) approach which is computed by integrating the electronic current passing through interatomic surfaces located by the atom-in-molecule theory (QTAIM) between neighboring atoms by using the AIMALL suite of programs.^56^ The purpose of the latter computations is to assess the aromatic character of the Al_13_^−^, Al_12_Si, and Al_11_Si_2_^+^ species. The magnetic current density derived from the MICD method is performed with the magnetic field vector perpendicular the molecular plane and out of that plane. As for a convention, clockwise flow of the magnetic current stands for a diamagnetic current, and anticlockwise flow for a paramagnetic current.

## 3. Results and discussion

### 3.1 Lower-lying isomers of Al_*n*_Si_*m*_ clusters in both neutral and cationic states

**Fig. 1:**
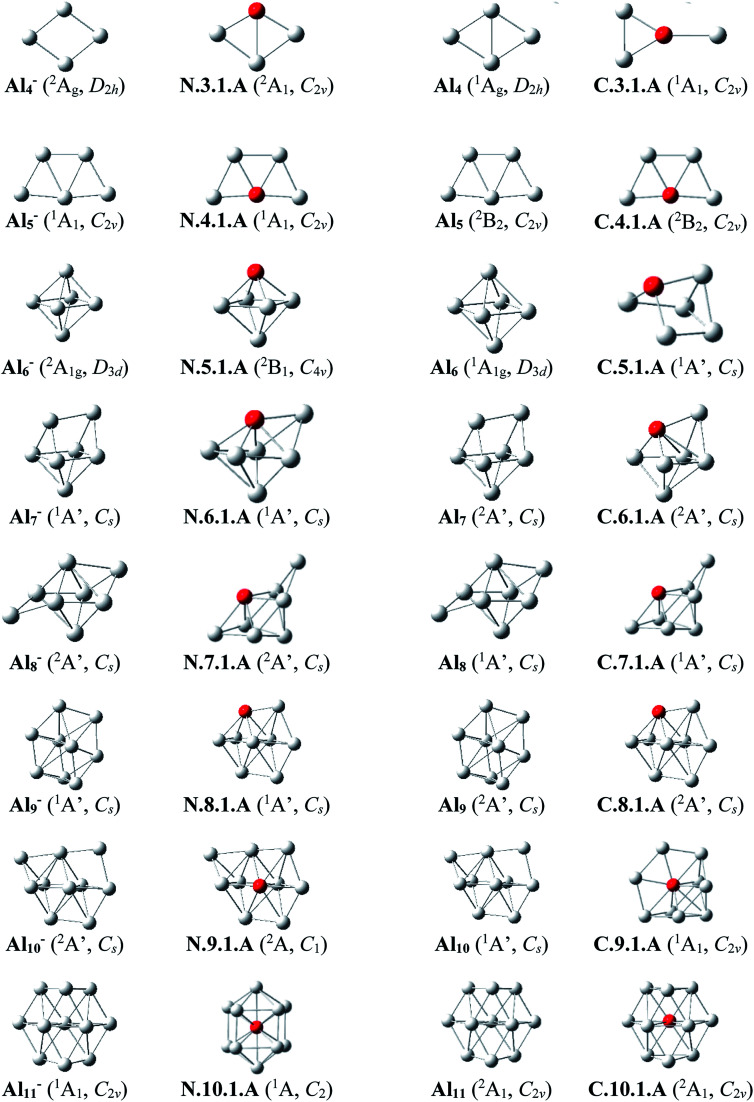
The shapes and electronic states of the lowest-lying isomers Al_*n*_Si with *n* = 3–10 and the global minimum of Al_*n*+1_ at the neutral and cationic states for Al_*n*_Si, corresponding to the anionic and neutral states for Al_*n*+1_, respectively.

**Fig. 2:**
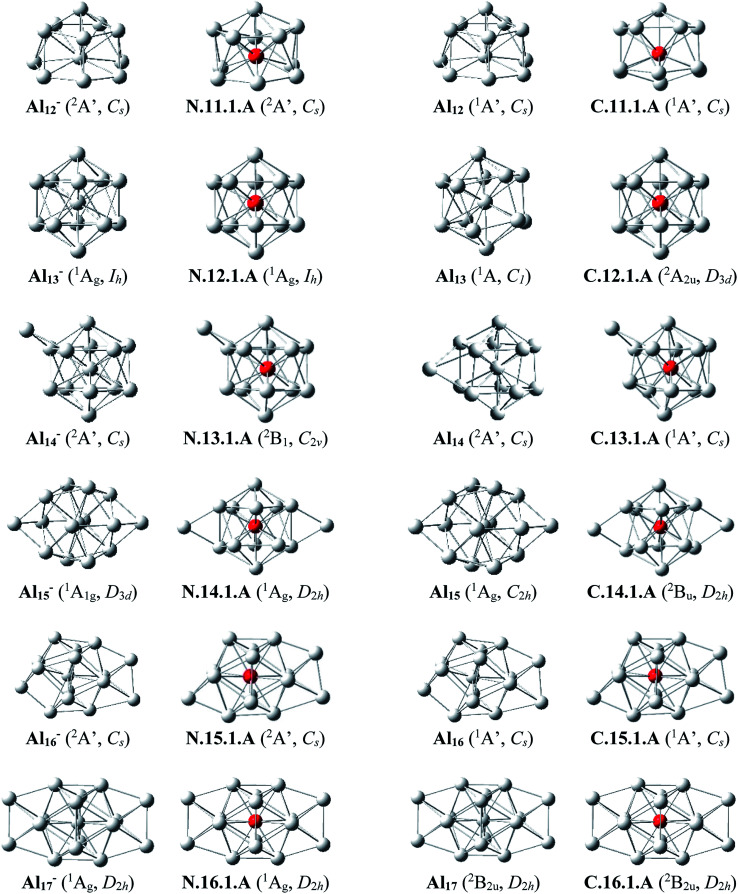
The shapes, electronic states of the lower-lying isomers Al_*n*_Si with *n* = 11–16 at the neutral and cationic states for Al_*n*_Si, corresponding to the anionic and neutral states for Al_*n*+1_, respectively.

**Fig. 3:**
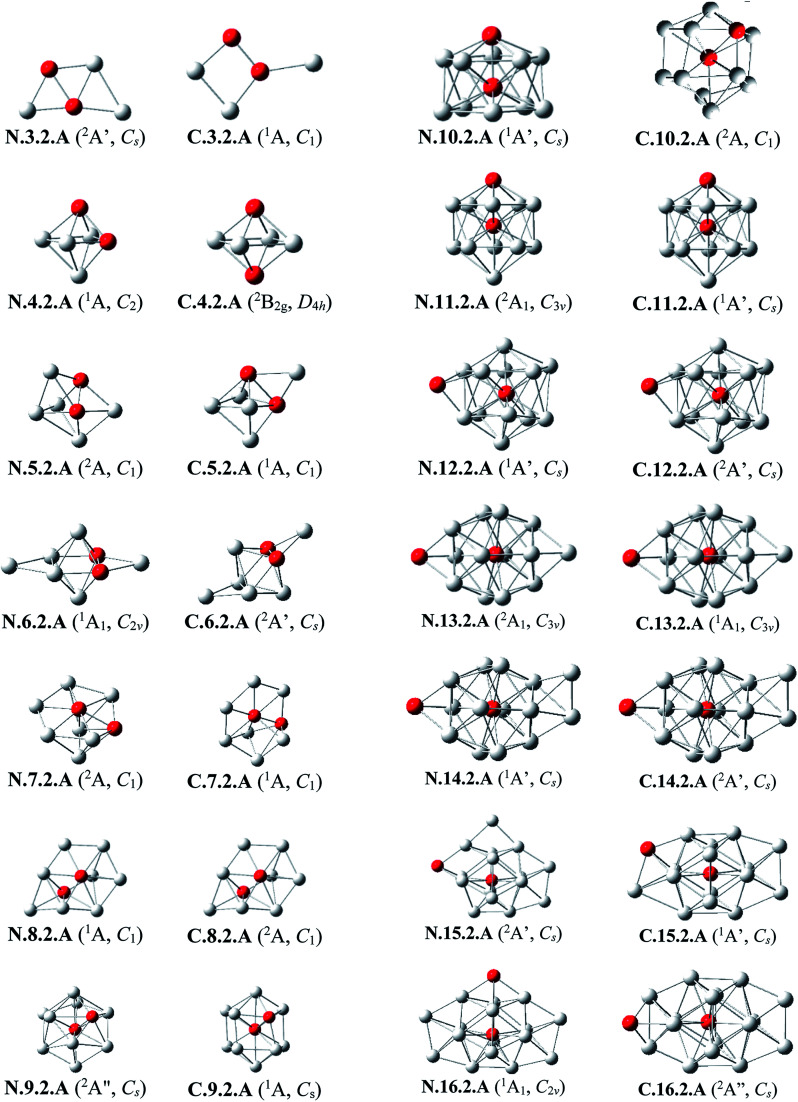
The shapes and electronic states of the lowest-lying isomers Al_*n*_Si_2_ with *n* = 3–16 at the neutral and cationic states. (a) Average binding energy (*E*_b_, eV) of the neutral Al_*n*_, Al_*n*_Si, and Al_*n*_Si_2_ computations. (b) Average binding energy (*E*_b_, eV) of the cationic Al_*n*_^+^, Al_*n*_Si^+^, and Al_*n*_Si_2_^+^.

Since there is a large number of isomers located on the potential energy surface of each size considered, we only present here some of the lowest-lying isomers whose relative energies are close to the corresponding ground state structure (<1.5 eV in relative energy). The shapes of the most stable Al_*n*_Si_*m*_ equilibrium structures at both neutral and cationic states in comparison to the ground states of the pure aluminum clusters Al_*n*+*m*_ having the same number of electrons, their symmetry point groups are shown in Fig. 1–3. Moreover, the lower-lying isomers and their relative energies obtained at the CCSD(T)/cc-pvTZ + ZPE level are shown in Fig. S1 and S2 of the ESI.†

As for a convention, label **X.*n*.*m*.Y** is used to denote the isomers in which **X** = **N** and **C** stands for a neutral and cationic state, respectively, ***n*** is the number of Al atoms, ***m*** is the number of Si atoms, and **Y** = **A**, **B**, **C**… referring to the different isomers with increasing relative energy. Accordingly, the notation **X.*n*.*m*.A** invariably refers to the most stable isomer **A** of the **X.*n*.*m*** series.

#### 3.1.1. Singly silicon doped Al_*n*_Si^0/+^

The most stable structures of the singly doped clusters are displayed in Fig. 1 (*n* = 3–10) and 2 (*n* = 11–16). Their main characteristics can be summarized as follows:

##### Al_3_Si

(U)CCSD(T) results emphasize that both isomers N.3.1.A and N.3.1.B are basically degenerate within an energy separation of only 0.06 eV. Unlike the neutral Al_3_Ti which favors a tetrahedral configuration due to the effect of the 3d orbital of Ti dopant,^25^ both are formed by substitution of an Al position of the planar neutral Al_4_ by an Si atom.^13^ Although the isomer N.3.1.C, formed by adding an Al^+^ cation on the Si-top of the triangular Al_2_Si, is 0.38 eV less stable than N.3.1.A, its corresponding cation, however, becomes the ground state C.3.1.A, with 0.32 eV lower in energy than the next isomer C.3.1.B.

##### Al_4_Si

Contrary to Si_4_Al having a three-dimensional shape due to the dominance of sp^3^ hybridization of Si atoms, lower-lying isomers of neutral Al_4_Si still keep the planar forms in their low-spin state. In n.4.1.A Si takes the place of central Al atom in the neutral Al_5_ (ref. 13) and connects with four remaining Al atoms. Similarly, C.4.1.A which is the corresponding cation of N.4.1.A, remains the most stable cationic isomer Al_4_Si^+^.

##### Al_5_Si

The most stable isomer N.5.1.A is generated by substitution of one Al atom in the octahedron of Al_6_ (ref. 13) by Si. The next isomer N.5.1.B in which Si replaces an Al position of a triangular prism in Al_6_,^9^ is much less stable than N.5.1.A by 0.40 eV. However, geometry optimizations indicate that the structure N.5.1.A does not exist as a cationic minimum on the potential surface; C.5.1.A which is actually the corresponding cation of N.5.1.B, becomes the ground state of the cation Al_5_Si^+^. There is thus a reversed energy ordering upon ionization.

##### Al_6_Si–Al_9_Si

For the singly doped Al_*n*_Si with *n* = 6–9, their geometries are mostly based on the pseudo-octahedron Al_5_Si in which the Si atom is placed at a top. Accordingly, the lowest-lying isomers for Al_6_Si, Al_7_Si, Al_8_Si and Al_9_Si in both neutral and cationic states have structural characteristics of Al_5_Si with capping of one, two, three, and four Al atoms, respectively, on different positions of the pseudo-octahedron. (U)CCSD(T) calculations indicate an energetic degeneracy between the two most stable isomers of the neutral Al_6_Si and Al_9_Si with energy gaps of 0.08 and 0.04 eV, respectively.

The corresponding cations of N.6.1.A, N.7.1.A, and N.8.1.A remain also the lowest-lying isomers of the cationic counterparts. Again, calculated results show that the two most stable isomers exist as energetically degenerate for both cations Al_6_Si^+^ and Al_7_Si^+^ with energy gap of only 0.03 and 0.01 eV, respectively. For the cation Al_9_Si^+^, C.9.1.B which is the corresponding cation of N.9.1.A, now lies 0.17 eV higher in energy than the *C*_2v_ symmetric structure C.9.1.A although N.9.1.C is the corresponding neutral of C.9.1.A and is 0.40 eV less stable than N.9.1.A.

##### Al_10_Si

The first Si encapsulated in the Al framework occurs at *n* = 10; N.10.1.A appears as a doped cage in which Si is located inside a bicapped quadrangular prism Al_10_ cage. The next isomer N.10.1.B, being 0.32 eV less stable, has the shape of pure Al_11_ cage^13^ in which one Al position is substituted by a Si atom. For the cationic state, the most stable isomer C.10.1.A formed by replacement of the Al atom located at the centre of the hexagonal face of pure Al_11_ by a Si atom. According to geometry optimization, a structure having the form of C.10.1.A does however not exist as a neutral minimum on the potential energy surface. Thus the C.10.1.A is presumably formed through a rearrangement upon ionization of a neutral Al_10_Si.

##### Al_11_Si

The most stable neutral N.11.1.A is obtained by substitution of an Al atom located at the centre of the pure Al_13_ icosahedral framework by Si and simultaneous removal of another Al from it. For the cation Al_11_Si^+^, (U)CCSD(T) calculations indicate that the two endohedral structures, C.11.1.A and C.11.1.B are practically degenerate with a negligible energy gap of 0.01 eV.

##### Al_12_Si–Al_16_Si

The icosahedral growth pattern appears to dominate the structural motifs of Al_*n*_Si^0/+^ clusters for the sizes *n* = 12–16. The global minima of both neutral and cationic Al_12_Si^0/+^ possess the popular icosahedral shape in which the Si atom is centered and connects with 12 Al atoms of the icosahedron cage Al_12_, and each is at ∼0.6 eV more stable than the next isomer. Based on that finding, the most stable structures of neutral clusters Al_13_Si, Al_14_Si, Al_15_Si and Al_16_Si are obtained by successive capping of one, two, three, and four Al atoms on different positions of the icosahedral Al_12_Si, and their corresponding cations remain the global minima of the positively charged state.

For the neutral Al_14_Si, calculations continue to emphasize an energetic degeneracy with an energy gap of 0.03 eV between the high symmetry structure N.14.1.A (*D*_2h_) and N.14.1.B in which a Si and an Al atom are exohedrally attached on the icosahedron Al_13_. In other words, it can be considered that N.14.1.B is formed by substituting a Si atom at an Al-top of the high symmetrical (*D*_3d_) anion Al_15_^−^ and this may lead to the stability of N.14.1.B.^12^ The Al_*n*_Si can be regarded to be generated upon substitution of the central Al position of the icosahedron Al_13_ of the Al_*n*+1_ species, with *n* = 12–16, by a Si atom.^12^

Generally, since the Si element has one valence electron more than the Al, the neutral Si is isoelectronic with the anion Al^−^. Therefore, it is observed in Fig. 1 and 2 that in small neutral Al_*n*_Si clusters (*n* ≤ 9), the Si atom prefers a substitution at an Al position, which exhibits a higher coordination number, of the pure aluminum framework of the isoelectronic anion Al_*n*+1_^−^. The neutral Al_10_Si represents however an exception because its most stable structure significantly differs from the Al_11_^−^ framework. The ground state structure of a cationic Al_*n*_Si^+^ cluster turns out to be also quite similar to those of the pure neutral Al_*n*+1_. Moreover, for the cluster Al_*n*_Si with *n* ≥ 10, the Si dopant prefers to be located at the inner cage formed by Al atoms. According to Weigend *et al.*,^57^ this can be rationalized by the fact that the Si atom has larger nuclear charge as compared to Al atom, and its valence orbitals (3s, 3p) are lower-lying in energy, more compact to its nucleus as compared to Al atom, causing a higher electron density at the center as compared to that in the surface of the Al_*n*_Si cluster.

#### 3.1.2. Doubly silicon doped Al_*n*_Si_2_^0/+^

**Fig. 4:**
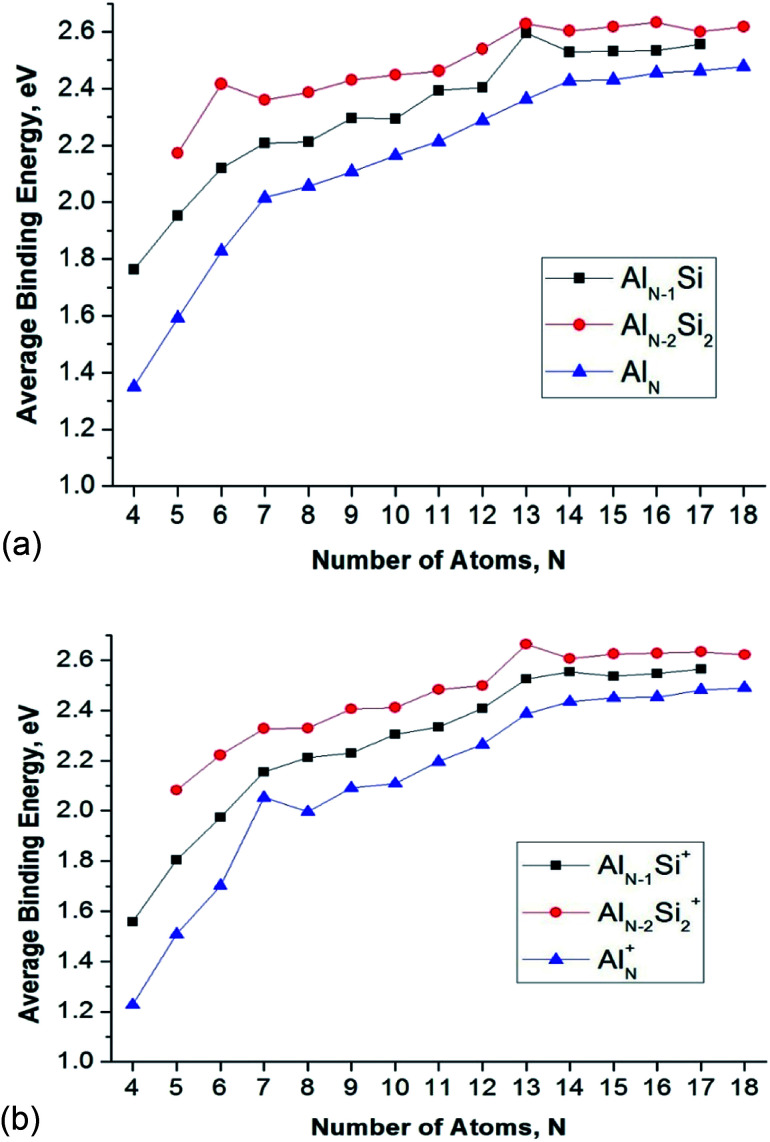
Average binding energy (*E*_b_, eV) of the Al_*n*_Si_*m*_/^0/+^ (*n* = 3–16, *m* = 1–2) clusters using (U)CCSD(T)/cc-pVTZ + ZPE. (a) MO diagram of Al13- anionic cluster. (b) MO diagram of Al_12_Si neutral cluster. (c) MO diagram of Al_11_Si_2_^+^ anionic cluster.

The main structural characteristics for these systems are displayed in Fig. 3 and 4.

##### Al_3_Si_2_

The lowest-lying isomers of both neutral and cationic states of Al_3_Si_2_ are characterized by a planar structure. N.3.2.A in which both Si atoms replace two Al positions in the neutral Al_5_ is the most stable isomer. According to geometry optimizations, the shape of N.3.2.A does not exist as a minimum on the potential energy surface of Al_3_Si_2_^+^. For the cation Al_3_Si_2_^+^, both isomers C.3.2.A and C.3.2.B are degenerate within a tiny energy gap of 0.07 eV. Both cationic structures are formed by adding an Al atom on a Si-top of the rhombic Al_2_Si_2_.

##### Al_4_Si_2_

The most stable structures N.4.2.A and N.4.2.B are obtained by substitution of two Al atoms in the octahedron of Al_6_ by two Si. Again, the cation having the shape of N.4.2.A does not exist as a cationic energy minimum. The high symmetry (*D*_4h_) isomer C.4.2.A generated by ionization of N.4.2.B emerges as the most stable cation.

##### Al_5_Si_2_–Al_8_Si_2_

Similar to the singly doped Al_*n*_Si with *n* = 6–9, for the Al_*n*_Si_2_ clusters consisting of a total number of seven to ten atoms, their geometrical structures are based on the octahedron Al_4_Si_2_ in which two Si atoms replace two Al positions of the pure octahedron Al_6_. The most stable isomers for Al_5_Si_2_, Al_6_Si_2_, Al_7_Si_2_ and Al_8_Si_2_ in both neutral and cationic states are thus obtained by capping of one, two, three and four Al atoms, respectively, on different positions of Al_4_Si_2_. Due to the presence of two Si dopant atoms, the octahedron Al_4_Si_2_ is however slightly distorted within a few Al_*n*_Si_2_ structures. (U)CCSD(T) calculations point out again an energetic degeneracy in several sizes including Al_5_Si_2_, Al_6_Si_2_, Al_6_Si_2_^+^, Al_7_Si_2_^+^ and Al_8_Si_2,_ with the largest energy gap of only 0.08 eV between the two corresponding lowest-lying isomers.

##### Al_9_Si_2_

From size *n* = 9, the doubly Si doped Al cluster tends to favor an icosahedral structure. N.9.2.A is accordingly formed following substitution of an external Al atom by a Si atom and simultaneous removal of two other Al atoms of the icosahedron Al_12_Si in which the Si atom is situated at its central position. For the cation, due to the fact that they have the same number of atoms and valence electrons, the most stable C.9.2.A has a similar shape to that of the ground state of neutral Al_10_Si N.10.1.A in which the additional Si atom now replaces an Al position.

##### Al_10_Si_2_

(U)CCSD(T) results consistently point out a degeneracy in energy between both isomers N.10.2.A and N.10.2.B for the neutral Al_10_Si, and among C.10.2.A, C.10.2.B, and C.10.2.C for the cation, with tiny energy gaps of 0.01 and 0.06 eV, respectively. Both neutrals N.10.2.A and N.10.2.B are generated from the same motif of Al_9_Si in which an Al atom is replaced by a Si atom and another Al atom is extracted from the icosahedral Al_12_Si. The cationic isomer C.10.2.A is formed by addition of an Al atom into the cation Al_9_Si_2_^+^C.9.2.A, isomer C.10.2.B is obtained by capping a Si atom into the neutral Al_10_Si, and isomer C.10.2.C is the corresponding distorted cation of N.10.2.A.

##### Al_11_Si_2_

Again, the icosahedral growth pattern prevails in generating the structures of Al_*n*_Si_2_ clusters for *n* = 11–16. Both the neutral and cationic most stable isomers Al_11_Si_2_^0/+^, N.11.2.A and C.11.2.A, prefer an icosahedral structure in which one Si atom is located at the central position while the remaining Si substitutes an external Al position of the icosahedron Al_13_ and they have a remarkably high thermodynamic stability in comparison to the remaining isomers.

##### Al_12_Si_2_

Both isomers N.12.2.A and N.12.2.B are found to exist as two degenerate equilibrium ground states for the neutral Al_12_Si_2_ with a tiny energy difference of 0.05 eV. N.12.2.A is obtained by capping a Si atom onto a surface of the icosahedron Al_12_Si framework, whereas N.12.2.B is generated upon capping of an Al atom onto an exohedral Al–Si edge of the icosahedron Al_11_Si_2_. Similarly, their corresponding cations, namely C.12.2.A and C.12.2.B, are also energetically degenerate with an even smaller energy separation of 0.01 eV.

##### Al_13_Si_2_–Al_16_Si_2_

For *n* = 13–16, the lowest-lying structures in both neutral and cationic states are constructed by preserving the icosahedron Al_12_Si skeleton and by adding the second Si atom and remaining Al atoms onto its different external positions. The global minima of Al_13_Si_2_, Al_14_Si_2_ and their corresponding cation have a similar geometric shape. For both neutral and cationic states of Al_15_Si_2_ and Al_16_Si_2_, (U)CCSD(T) calculations result again in energetic degeneracies of the two lowest-lying isomers, with energy gap <0.1 eV.

Similar to the growth pattern of singly silicon doped Al_*n*_Si^0/+^, it can be considered that the structures of doubly silicon doped Al_*n*_Si_2_^0/+^ are obtained following substitution at two Al positions of the pure corresponding aluminum framework Al_*n*+2_ by two Si atoms. For *n* = 12–16, Al_*n*_Si_2_^0/+^ are built up on the basis of an icosahedral growth path in which one Si atom is situated at the centre of the icosahedral Al_12_Si, whereas the additional Si atom is now externally attached.

### 3.2 Relative stability of clusters

In order to evaluate the inherent thermodynamic stability of the clusters considered, the average binding energies (*E*_b_) are determined and compared to those of the relevant pure aluminum clusters. The average binding energies (*E*_b_) can conventionally be defined as follows (eqn (1)–(6)):

1 *E*_b_(Al_*n*_Si) = [*nE*(Al) + *E*(Si) − *E*(Al_*n*_Si)]/(*n* + 1)

2 *E*_b_(Al_*n*_Si^+^) = [(*n* − 1)*E*(Al) + *E*(Al^+^) + *E*(Si) − *E*(Al_*n*_Si^+^)]/(*n* + 1)

3 *E*_b_(Al_*n*_Si_2_) = [*nE*(Al) + 2*xE*(Si) − *E*(Al_*n*_Si_2_)]/(*n* + 2)

4 *E*_b_(Al_*n*_Si_2_^+^) = [(*n* − 1)*E*(Al) + *E*(Al^+^) + 2*xE*(Si) − *E*(Al_*n*_Si_2_^+^)]/(*n* + 2)

5 *E*_b_(Al_*n*_) = [*nE*(Al) − *E*(Al_*n*_)]/*n*

6 *E*_b_(Al_*n*_^+^) = [(*n* − 1)*E*(Al) + *E*(Al^+^) − *E*(Al_*n*_^+^)]/*n*

where *E*(Si), *E*(Al), and *E*(Al^+^) are the total energies of the Si-atom, Al-atom and the cation Al^+^, respectively. Since the ionization energy of Al atom (1.56 eV) is lower than Si atom (2.1 eV), the total energies of the cation Al^+^ is thus using to calculate the average binding energy instead of the total energies of the cation Si^+^. For their part, *E*(Al_*n*_Si), *E*(Al_*n*_Si^+^), *E*(Al_*n*_Si_2_), *E*(Al_*n*_Si_2_^+^), *E*(Al_*n*_), and *E*(Al_*n*_^+^) are total energies of the neutral Al_*n*_Si, cationic Al_*n*_Si^+^, neutral Al_*n*_Si_2_, cationic Al_*n*_Si_2_^+^, neutral Al_*n*_, and cationic Al_*n*_^+^ structures, respectively. All energetic values are obtained at CCSD(T)/cc-pvTZ with ZPE corrections. Geometrical parameters of the pure aluminum clusters at both neutral and cationic states are obtained from the previous studies.^11,13^ Where needed, geometries are reoptimized using (U)B3LYP/6-311+G(d) calculations. The plots illustrating the *E*_b_ evolution are displayed in Fig. 4.

The *E*_b_ value tends to rise with increasing cluster sizes. For singly doped Al_*n*_Si clusters, the neutral Al_12_Si reveals the largest *E*_b_ value as compared to the remaining singly doped neutral species. Of the neutral Al_*n*_Si_2_ clusters, Al_4_Si_2_ presents an enhanced *E*_b_ value as compared to those of the remaining doubly doped species. In the series of cations considered, Al_11_Si_2_^+^ gets a maximum peak in the *E*_b_ plot which demonstrates its higher thermodynamic stability.

The enhanced relative stability of these clusters can simply be rationalized in terms of “magic clusters” in which the number of valence electrons amounts to 2, 8, 18, 20, 34, 40 and 58… *etc.* Accordingly, the closed electron shells [1S^2^ 1P^6^ 1D^10^ 2S^2^ 1F^14^ 2P^6^…] of the neutral Al_4_Si_2_ contains the same number of 20 valence electrons but an atom fewer in comparison to the pure cation Al_7_^+^, while both Al_12_Si and Al_11_Si_2_^+^ have each 40 valence electrons and the same icosahedral structure with a total number of 13 atoms such as the anion Al_13_^−^. As a result, they behave as “magic clusters” with an enhanced thermodynamic stability.

Fig. 4 also displays a comparison of the binding energies of the pure Al_*n*_, Al_*n*_Si, and Al_*n*_Si_2_ using (U)CCSD(T)/cc-pVTZ + ZPE calculated results in which the *E*_b_ value of Al_*n*_Si is larger than the *E*_b_ value of Al_*n*_ and smaller than Al_*n*_Si_2_ at the same number of atoms. Accordingly, it appears that the Si dopant tends to increase the cluster stability with respect to various fragmentation processes.

### 3.3 Adiabatic ionization energies

Calculated total energies carried out at the (U)CCSD(T) level are now used to evaluate the adiabatic ionization energies (AIE), that are obtained from differences between total energies of the neutrals Al_*n*_Si_*m*_ and their corresponding Al_*n*_Si_*m*_^+^ cations. In general, the AIE values are rather low, but there is no consistent trend in going from one dopant to two dopants. In view of the frequent energetic degeneracy of many neutral sizes, Table 1 displays the AIE values derived from the two most stable isomers Al_*n*_Si_*m*_ and these values are compared to those of pure aluminum clusters. Due to the close AIE values, the corresponding photoelectron (PE) spectra of these neutrals are expected to be rather complicated having broad bands. Only an appropriate treatment considering the vibrational progressions upon ionization can usefully predict their PE spectra. Let us note that both “magic clusters” Al_4_Si_2_ and Al_12_Si are remarkably characterized by the largest AIE values, being 7.1 and 6.9 eV, respectively, that are no doubt due to an enhanced stability of these neutrals.

**Table 1:** Adiabatic Ionization Energies (AIE, eV) of singly and doubly silicon doped aluminum clusters in comparison with pure aluminum clusters Al_*n*_ (CCSD(T) calculations)

Number of atoms, *k*	Al_*k*−1_Si	Al_*k*−2_Si_2_	Al_*k*_
	N.*k* − 1.1.A	N.*k* − 2.2.A	
4	6.75	—	6.43
5	6.67	6.39	6.36
6	6.80	7.10	6.69
7	6.30	6.17	5.68
8	5.94	6.40	6.42
9	6.55	6.16	6.09
10	5.83	6.31	6.49
11	6.59	5.71	6.13
12	5.88	6.43	6.23
13	6.85	5.46	5.63
14	5.60	5.88	5.84
15	5.89	5.84	5.66
16	5.71	6.03	5.97
17	5.79	5.36	5.63
18	—	5.87	6.05

### 3.4 Dissociation energies

To evaluate further the thermodynamic stability, the dissociation energies (*D*_e_) for the various fragmentation channels of the clusters considered are determined. Results calculated from (U)CCSD(T) energies are listed in Table 2.

**Table 2:** Dissociation energies (*D*_e_, eV) for various fragmentation channels of Si_*n*_Al_*m*_ (*n* = 3–16, *m* = 1–2) from CCSD(T)/cc-pVTZ calculations

*n*	*D*_e_(1) remove a Si				*D*_e_(2) remove a Al				*D*_e_(3) remove a Si^+^		*D*_e_(4) remove a Al^+^	
	Al_*n*_Si	Al_*n*_Si^+^	Al_*n*_Si_2_	Al_*n*_Si_2_^+^	Al_*n*_Si	Al_*n*_Si^+^	Al_*n*_Si_2_	Al_*n*_Si_2_^+^	Al_*n*_Si^+^	Al_*n*_Si_2_^+^	Al_*n*_Si^+^	Al_*n*_Si_2_^+^
3	3.56	3.22	3.82	4.18	n/a	n/a	n/a	n/a	4.91	5.53	n/a	n/a
4	4.36	4.12	4.74	4.31	2.71	2.79	3.63	2.92	5.79	5.74	1.98	2.47
5	4.75	4.31	3.81	4.44	2.95	2.82	2.02	2.96	6.06	5.75	2.09	1.79
6	4.49	4.88	3.64	3.54	2.74	3.24	2.57	2.34	6.29	5.35	2.38	2.11
7	3.59	3.33	4.17	3.95	2.25	2.61	2.77	3.02	5.76	6.12	2.25	2.56
8	4.22	4.09	3.81	4.06	2.97	2.36	2.62	2.46	5.77	5.61	2.36	2.25
9	3.97	4.23	4.16	4.27	2.26	2.99	2.61	3.2	6.25	6.55	2.37	2.83
10	4.68	4.58	4.16	4.32	3.39	2.62	3.39	2.67	6.2	5.83	2.73	2.9
11	4.49	4.75	5.32	5.73	2.53	3.24	3.68	4.65	6.72	7.96	2.59	4.16
12	6.28	5.66	2.7	3.67	4.9	3.93	2.28	1.86	7.53	4.92	3.99	2.34
13	4.69	4.72	3.87	3.63	1.66	2.91	2.83	2.88	7.2	6.14	2.00	2.93
14	4	3.94	4.15	4.01	2.58	2.29	2.86	2.67	6.22	6.23	2.63	2.77
15	4.07	4.01	3.66	4.02	2.55	2.73	2.06	2.74	6.46	6.41	2.78	2.64
16	4.19	4.37	3.66	3.59	2.93	2.85	2.93	2.42	6.5	5.9	3.08	3.01

The *D*_e_ values of the neutrals Al_*n*_Si for the Si removal channel (1) Al_*n*_Si → Al_*n*_ + Si turn out to be larger than those for the Al-loss channel (2) Al_*n*_Si → Al_*n*−1_Si + Al proving the stronger Si–Al bond. From the obtained knowledge in solid state, the loss of Al is a lower energy channel than the loss of Si, and this can be understood by the fact that the cohesive energy of Si (4.63 eV per atom) is larger than that of Al (3.39 eV per atom). Similar observations can be made for the positively charged species in that Al_*n*_Si^+^ tend to be fragmented generating one Al element or its cation Al^+^ plus a smaller Al_*n*−1_Si^+/0^ along the fragmentation channels (2) and (4).

Again, for doubly doped neutral Al_*n*_Si_2_ clusters, dissociation energies for Si-loss channels (1) Al_*n*_Si_2_ → Al_*n*_Si + Si are constantly larger than those for Al-elimination pathways (2) Al_*n*_Si_2_ → Al_*n*−1_Si_2_ + Al. Similarly, the cations Al_*n*_Si_2_^+^ follow a preferential fragmentation to form one Al^0/+^ plus a smaller Al_*n*−1_Si^+/0^ as described in the channels (2) and (4) (*cf.*Table 2).

Moreover, from the values given in Table 2, we can infer two exchange reactions:

R1 Al_*n*_ + Si → Al_*n*−1_Si + Al;

and

R2 Al_*n*_Si + Si → Al_*n*−1_Si_2_ + Al

The energies the exchange reaction (R_1_) and (R_2_) can easily be calculated *via* the differences between *D*_e_(1) and *D*_e_(2) of Al_*n*_Si and Al_*n*_Si_2_. Generally, all energetic values of (R_1_) and (R_2_) are approximately in a range of 0.5–2 eV. Remarkably, the maximum value of 3.03 eV, obtained at *n* = 12 for (R_1_), corresponds to that of Al_12_Si. It proves the very particular case of neutral Al_12_Si for which the energy of (R_1_) is much higher than for any other case.

### 3.5 Electronic structures of the isoelectronic Al_13_^−^, Al_12_Si, and Al_11_Si_2_^+^ having 40 valence electrons

While a single atom possesses discrete electron energy levels and bulk solids have continuous energy levels, the electron energy levels of atomic clusters could be distributed in a non-uniform manner. Clusters having high symmetry frequently result in pronounced electron shells, such as 1S, 1P, 1D, 2S, 2P, 1F… This formation of electron shells is manifested in many physical properties of finite, quantal many-electron systems, such as, for example, their stability, ionization energies, chemical reactivity, or conductance, *etc.* A brief review of the abundant literature on the phenomenon reveals that both cationic Al_7_^+^ and anionic Al_13_^−^ aluminum clusters (Scheme 1) behave as “magic clusters” with enhanced thermodynamic stability, in part due to the fact that they possess the closed electron shells including 20 and 40 valence electrons, respectively. As stated above, the neutral Al_13_ appears as a ‘superhalogen’ characterized by a very large electron affinity exceeding those of halogen atoms.^8,15,58–60^

Of the whole series of the Al_*n*_^−^, Al_*n*−1_Si, and Al_*n*−2_Si_2_^+^ clusters, let us consider a typical series of high symmetry isoelectronic structures Al_13_^−^, Al_12_Si and Al_11_Si_2_^+^ to emphasize on how an assemblage of degenerate or nearly degenerate levels is manifested in their optical absorption spectra.

**Fig. 5:**
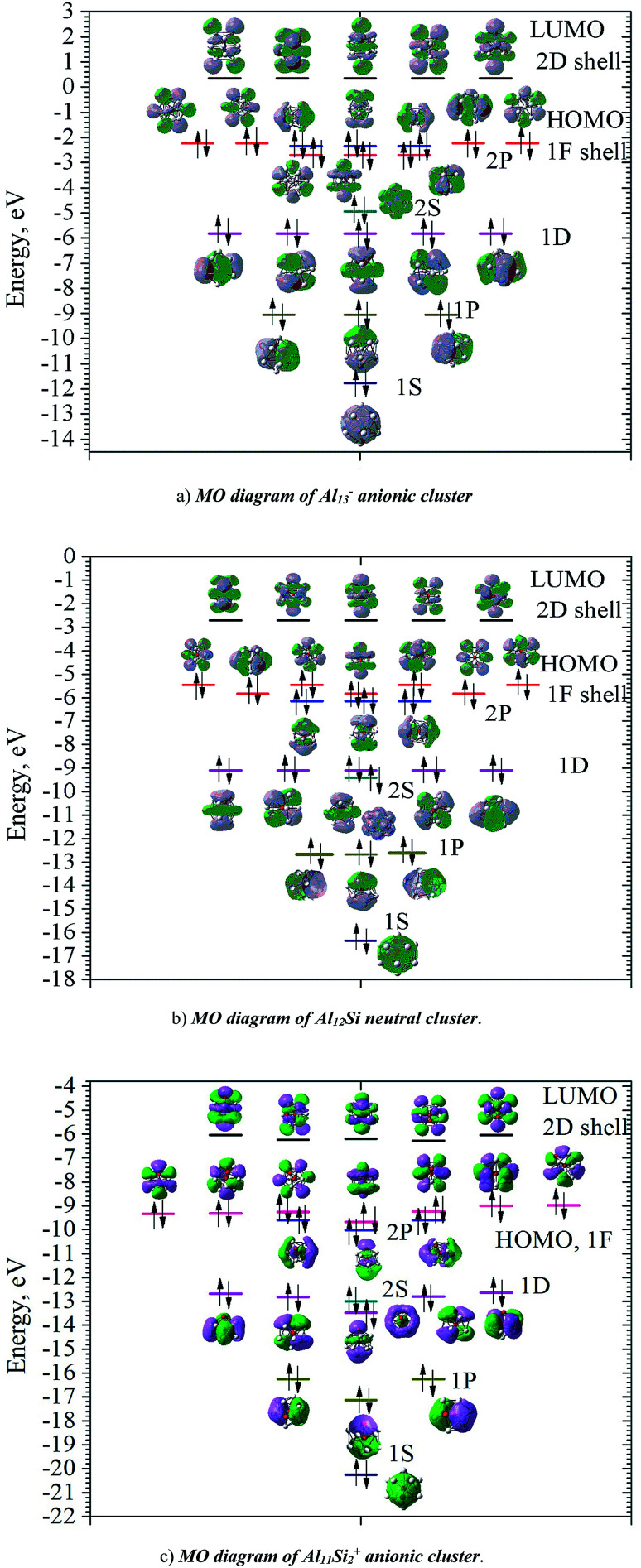
MO diagram of Al_13_^−^, Al_12_Si, and Al_11_Si_2_^+^ cluster. Black lines represent LUMO; the filled pink, blue, dark cyan, magenta, dark yellow and navy lines represent orbitals corresponding to the 1F, 2P, 2S, 1D, 1P, and 1S shells, respectively. Black arrows represent electrons (alpha spin: up arrow; beta spin: down arrow).

In order to investigate the electronic structure of Al_13_^−^, Al_12_Si, Al_11_Si_2_^+^, their MO diagrams are first plotted and displayed in Fig. 5. The electron configuration of the outer electron shell of each cluster could then be written as […1S^2^ 1P^6^ 1D^10^ 2S^2^ 2P^6^ 1F^14^…], in which the high fold degeneracy levels such as the 1F, 1D, even the 1P and 2P could be split and mixed with other levels. To confirm that the number of 40 electrons in each cluster shell are arising from the valence electrons of Al atoms, we calculate the contributions of atomic orbitals (AO) of Al atoms to the shell MOs by using Natural Bonding Orbital (NBO) analysis.

The contributions of s, p and d AOs of Al atoms into the shell MOs of the anion Al_13_^−^ are found as follows: 1S = 94% s + 5% p + 0.1% d; 1P = 81% s + 18% p + 0.22% d; 1D = 89% s + 10% p + 0.49% d; 2S = 97% s + 3% p + 0.25% d; 1F (three lower 1F shell orbitals) = 94% s + 5% p + 0.22% d; 2P = 10% s + 89% p + 0.74% d and 1F (four higher ones) = 0% s + 98% p + 1.6% d. This result shows that the lower shell orbitals 1S, 1P, 1D, 2S, 1F (three lower ones) are mainly constructed from the s(Al) AOs and the higher shell orbitals 2P and 1F (four higher orbitals) are mainly constructed from p(Al) AOs. In other words, the 26 s-electrons of 13 Al atoms form the lower shells [1S^2^ 1P^6^ 1D^10^ 2S^2^ 1F^6^…] and 14 p-electrons including the adding one (to form the negative charge) form the higher shell [2P^6^ 1F^8^]. It is worth to note that while for the single Al atom, the 3s–3p energy gap is 3.60 eV,^50^ in the Al_13_^−^ cluster, according to our calculations, the gap between the 1F lower shell, which is formed from 3s(Al) AOs and the 2P shell which is formed from 3p(Al) AOs, is now reduced to 0.36 eV.

Similarly, the contributions of s, p and d AOs of Al and Si atoms into the shell MOs of Al_12_Si are found as follows: 1S = 90% s(Si) + 9% s(Al); 1P = 22% p(Si) + 12% p(Al) + 66% s(Al); 2S = 96% s(Si) + 4% s(Al); 1D = 8% p(Al) + 91%s(Al); 2P = 40% p(Si) + 8% p(Al) + 52% s(Al); 1F = 6% p (Al) + 94% s(Al) (three lower orbitals); and 1F = 2% d(Al) + 98% p(Al) (four higher orbitals). Introduction of a Si atom into the center of Al_12_ also splits the 1F level into two sub-levels in which one sub-level has four-fold degeneracy (higher 1F) and the other is three-fold (lower 1F), as it is shown in Fig. 5a and b. The energy gap between them is ∼0.47 eV for Al_13_^−^ and ∼0.36 eV for Al_12_Si. Unlike the 1F shell orbitals, the shells 1P, 1D and 2P do not split upon introduction of a Si atom into the center of the Al_12_ icosahedron. The shapes of higher 1F shell-orbitals (four-fold degeneracy) are similar to those of the 4f(*y*(3*x*^2^ − *y*^2^)), 4f(*yz*^2^), 4f(*xz*^2^) and 4f(*x*(3*y*^2^ − *x*^2^)), and the shapes of lower 1F shell orbitals are similar to those of 4f(*xyz*), 4f(*z*(*x*^2^ − *y*^2^)) and 4f(*z*^3^) AOs. The splitting of 1F shell orbitals described above can be understood on the basis that the central ions (Al^3+^ and Si^4+^) cause the orbitals with small angular momentum (they have larger densities in the inner part) more energetically favorable.

Herein the three lower 1F shell orbitals are constructed mainly from s(Al) AOs having small angular momentum, and the four higher 1F shell orbitals are composed of p(Al) AOs having larger angular momentum. It is also worth noting that in the formation of 2P shell orbitals, the 3p AOs of the Si atom contribute up to 40% and the contribution of 3s AOs of Al atoms drops to 52%, while in the formation of the three lower 1F shell orbitals, the 3s(Al) AOs are the dominant contributor (94%). This constitutes a reason that in the Al_12_Si cluster, the 2P orbitals are located below the three lower 1F orbitals as it is shown in Fig. 5b. Similarly, the MO diagram of the Al_11_Si_2_^+^, also having 40 valence electrons, is shown in Fig. 5c and described in detail in ESI.†

As for a comparison, orbital energies of the Al_13_^−^, Al_12_Si, and Al_11_Si_2_^+^ clusters are also illustrated in Fig. 5. Let us briefly state again that, for the Al_13_^−^ anion and the Al_12_Si neutral, owing to an icosahedral structure with *I*_h_ symmetry, orbital degeneracy is observed for each of the 1P, 1D shells, and the 1F shell which is split into two subshells. As compared to the case of neutral Al_12_Si cluster, orbital energy levels of the anion Al_13_^−^ are significantly higher due to the repulsion induced by the extra electron.

For Al_12_Si, the level ordering of 2S and 1D is reversed (relative to those in Al_13_^−^), and the 2P levels obviously get lower energy. For Al_11_Si_2_^+^, the 2P, 1D and 1F states markedly split due to the Si substitution and the lower symmetry, and a mixture of both 2P and 1F orbitals, as well as a mixture of the 2S level with the 1D levels are observed. However, the widths of the 1D/2S and 1F/2P sub-bands are still narrower than the spacings between other bands. In fact, this is the main feature for keeping the jellium model valid. The ellipsoidal shell structures in metal clusters were previously discussed by Brack and Clemenger^61,62^ and crossovers of the subshells in the deformed structures are common.

Calculated results also show that the clusters considered probably become semiconductors as suggested by the HOMO–LUMO energy gap ∼2.6 eV, though aluminum in its bulk form is a good conductor. The HOMOs of these clusters are the 1F and 2P shells, while the LUMOs are the 2D shells allow us to expect that electronic transitions from HOMO to LUMO is allowed *Δ* = ±1.

**Fig. 6:**
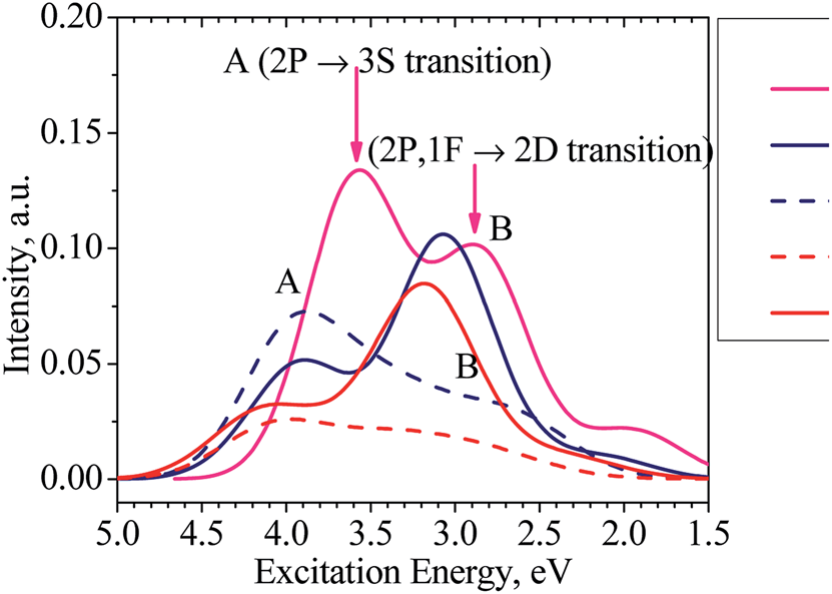
Absorption spectra of the clusters Al_13_^−^ (magenta line), Al_12_Si_1_^0^ (navy solid line for isomer N.12.1.A and navy dashed line for isomer N.12.1.B), and Al_11_Si_2_^+^ (red solid line for isomer C.11.2.A and red dashed line for isomer C.11.2.B).

Moreover, our TD-DFT calculations indicate that the absorption spectra of the clusters considered, as illustrated in Fig. 6 and listed in Table S1 in ESI,† have two main characteristic absorption bands in the visible region. The absorption spectrum of the Al_13_^−^ cluster is characterized by two strong bands which are centered at ∼345 (peak A) and ∼435 nm (peak B). The first peak emerges at ∼345 nm due to electronic transitions from 2P to 3S shells. The second band corresponds to electronic transitions collectively from the 1F shell orbital (HOMOs) as well as 2P shell orbitals to the 2D shell orbitals (LUMOs).

It is worth noting that the absorption spectra of small (*n* ≤ 5) Al_*n*_ clusters are dominated by discrete orbital transition, while for the larger size clusters, more collective electron transitions are responsible for their absorption spectra.^63^

The absorption spectra of both clusters Al_12_Si and Al_11_Si_2_^+^ are very similar to each other and characterized by two absorption bands centered at 316–403 and 403–394 nm, respectively. Analogous to the case of Al_13_^−^ anion, the longer wavelength absorption band at 403–394 nm is also due to electronic transitions collectively occurred from the 2P and 1F-the higher shell orbitals to the 2D shell orbitals. The 2P → 3S electronic transition is responsible for the shorter wavelength peak. As compared to the Al_13_^−^ anion, the corresponding absorption peaks of these two Si-doped clusters shift to the shorter wavelength (Fig. 6). This is reasonable because the metal Al atom(s) are substituted by the semi-metal Si atom(s) and the net charge of the cluster is changed from negative to neutral and positive values.

For the purpose of comparison, the absorption spectra of the second isomers of the Al_12_Si, isomer N.12.1.B, (*D*_5h_) and Al_11_Si_2_^+^C.11.2.B, (C_1_) clusters are also computed and plotted in Fig. 6. Similar to other isomers, the absorption spectra of N.12.1.B and C.11.2.B are also characterized by two absorption bands (A and B) in the visible region. For these isomers, the electronic transition from 1F → 2D (HOMO → LUMO) gives rise to a low intensity absorption band centered at around 450 nm. While the absorption band A remains almost unchanged (*cf.*Fig. 6), the absorption band B shifts from higher to lower intensity and from shorter to longer wave length, as compared to the isomers N.12.1.A and C.11.2.A. In view of the fact that N.12.1.B is 0.66 eV less stable than N.12.1.A and C.11.2.B is 0.43 eV higher in energy than C.11.2.A, their absorption bands (Fig. 6) are probably not observed, in case that the isomers are thermally populated. However, for other sizes where both lowest-lying isomers are energetically degenerate, each resulting UV-Vis absorption spectrum is likely produced by a superposition of both spectra of both corresponding **A** and **B** isomers.

**Fig. 7:**
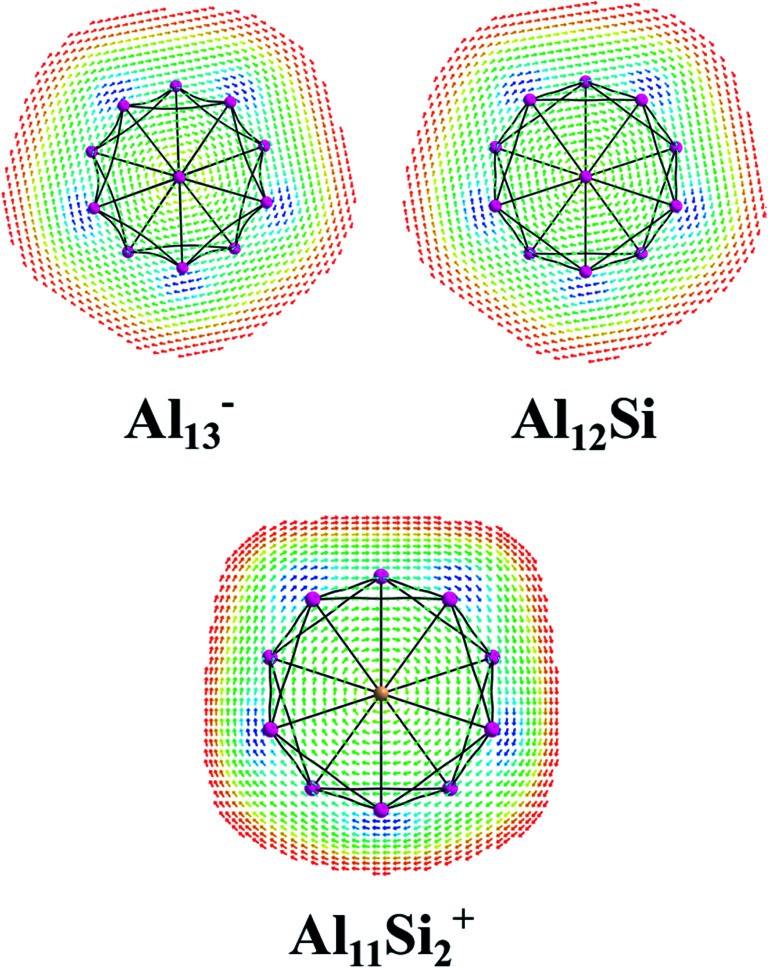
Profiles of current density for Al_13_^−^, Al_12_Si, and Al_11_Si_2_^+^ at the planes located above the center atoms by one Bohr. Red to blue arrows represents weak to strong current density with the range: 0 to 0.0006 a.u.

**Fig. 8:**
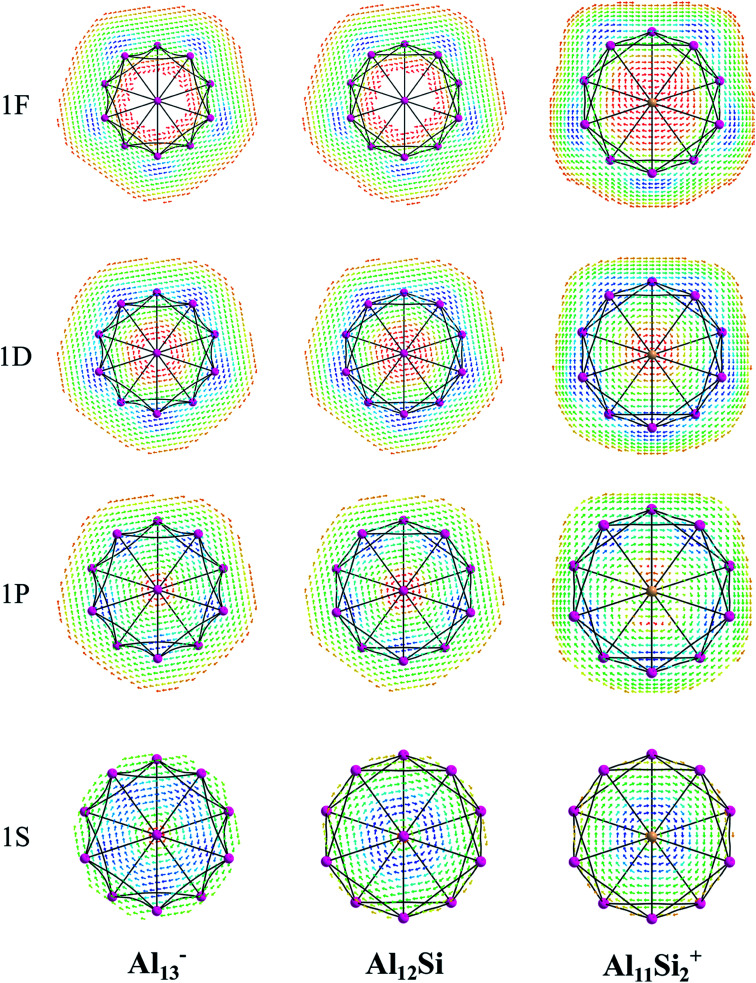
Profiles of current density for Al_13_^−^, Al_12_Si, and Al_11_Si_2_^+^ for the 1S, 1P, 1D, and 1F shell at the planes located above the center atoms by one Bohr. Red to blue arrows represents weak to strong current density.

**Fig. 9:**
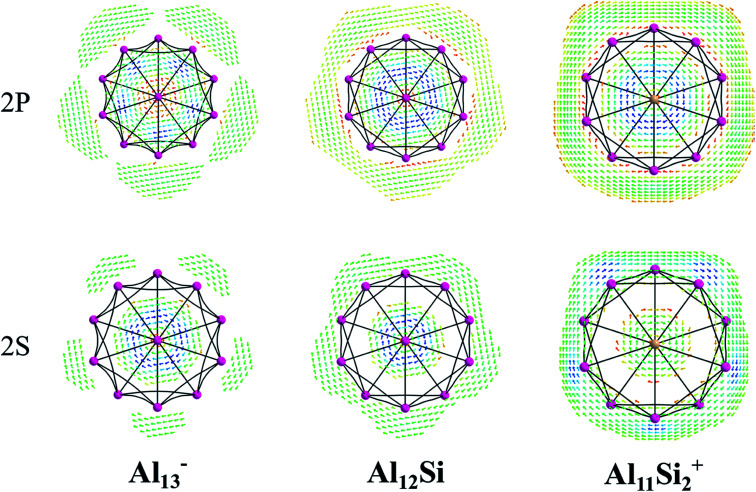
Profiles of current density for Al_13_^−^, Al_12_Si, and Al_11_Si_2_^+^ for the 2S, and 2P shell at the planes located above the center atoms by one Bohr. Red to blue arrows represents weak to strong current density.

The magnetic maps of Al_13_^−^, Al_12_Si and Al_11_Si_2_^+^, obtained by using the MICD approach, are shown in Fig. 7. The clockwise flows of the magnetic current point out that Al_13_^−^, Al_12_Si, and Al_11_Si_2_^+^ are aromatic structures thanks to the presence of 40 valence electrons. Furthermore, the MICD maps for each shell of Al_13_^−^, Al_12_Si and Al_11_Si_2_^+^, that are shown in Fig. 8 and 9, indicate that they are all aromatic. The blue arrows correspond to the strongest current density in each map. Fig. 8 displays the MICD maps for the 1S, 1P, 1D, and 1F shells of Al_13_^−^, Al_12_Si, and Al_11_Si_2_^+^. The positions of blue arrows in these structures move from the center to the outer of each structure when the electron shell change from the 1S to 1P to 1D and to 1F. The MICD maps confirm the validity of the shell model for these structures. As for a comparison, the MICD maps for both 2S and 2P shells are shown in Fig. 9. Obviously, the profiles of MICD maps for all the shells considered in Fig. 8 and 9 denote the electrons of 2S and 2P shells effect at both inside and outside the cage, whereas the electrons of 1S, 1P, 1D and 1F shells only induce effect within the cage of clusters.

## 4. Concluding remarks

In the present theoretical study, geometrical structures of lower-lying isomers of Al_*n*_Si_*m*_ clusters in both neutral and cationic states were investigated using DFT, TD-DFT with the (U)B3LYP/6-311+G(d) functional/basis set, and coupled-cluster theory (U)CCSD(T)/cc-pvTZ calculations.

All the doped clusters considered are characterized by low spin ground states, singlet for closed-shell species, and doublet for open-shell counterparts. For small size clusters, the Si atom prefers to be located at vertices having many edges. For larger sizes, the Si atom prefers to be endohedrally doped inside the corresponding Al_*n*_ cages.

A typical characteristic in most Si doped Al clusters is a consistent degeneracy of energies of the two lowest-lying isomers, either in the neutral or the cationic state. Such a characteristic suggests the coexistence of two distinct isomeric equilibrium structures, which is expected to give rise to complicated spectra, such as vibrational, electronic or photoelectron spectra.

Relative stabilities of the clusters considered, as compared to their neighbours, were also determined. Adiabatic ionization energy and dissociation energy of each cluster size were evaluated. Calculated results confirm the high stability of the Al_4_Si_2_, Al_12_Si and Al_11_Si_2_^+^ species as “magic clusters”, that are clusters having 20 or 40 valence electrons distributed over their outer electron shells.

The UV-Vis absorption spectra of the isoelectronic magic clusters Al_13_^−^, Al_12_Si, and Al_11_Si_2_^+^ have also been modelled, showing that they have two pronounced bands centered in the regions of ∼440 and ∼350 nm, corresponding to blue light and violet light, respectively. A rationalization for the absorption spectra was assisted by an analysis of the jellium shell model of the clusters involved. Moreover, MICD maps reveal that the isoelectronic series Al_13_^−^, Al_12_Si and Al_11_Si_2_^+^, having an aromatic shell, are aromatic structures possessing 40 valence electrons.

## Conflicts of interest

The authors declare no competing financial interest.

## Supplementary Material

RA-009-C9RA04004H-s001
