# Supplementary material for: Structure, stability, absorption spectra and aromaticity of the singly and doubly silicon doped aluminum clusters AlnSim0/+ with n = 3–16 and m = 1, 2
Source: RSC Adv. 2019 Aug 30;9(47):27208–23. doi: 10.1039/c9ra04004h (PMC9070575; doi:10.1039/c9ra04004h)
Supplement: RA-009-C9RA04004H-s001 [file RA-009-C9RA04004H-s001.pdf]

Electronic Supplementary Material (ESI) for RSC Advances.
This journal is © The Royal Society of Chemistry 2019

Structure, stability, absorption spectra and aromaticity of the singly and doubly silicon doped aluminum clusters $Al_nSi_m^{0/+}$ with n = 3 – 16 and m = 1, 2

Nguyen Minh Tam[a,b,]* Long Van Duong,[c] Ngo Tuan Cuong,[d] and Minh Tho Nguyen[e]

[a] *Computational Chemistry Research Group, Ton Duc Thang University, Ho Chi Minh City, Vietnam*

[b] *Faculty of Applied Sciences, Ton Duc Thang University, Ho Chi Minh City, Vietnam*

Email: nguyenminhtam@tdtu.edu.vn

[c] *Institute for Computational Science and Technology (ICST), Quang Trung Software City, Ho Chi Minh City, Vietnam*

[d] *Faculty of Chemistry and Center for Computational Science, Hanoi National University of Education, Hanoi, Vietnam*

[e] *Department of Chemistry, KU Leuven, Celestijnenlaan 200F, B-3001 Leuven, Belgium*

Supplementary Information

1. Shapes, electronic states and relative energies (ΔE, eV) of the lower-lying isomers Al_nSi_m at the neutral and cationic states. ΔE values are obtained from (U)CCSD(T)/cc-pVTZ + ZPE computations.

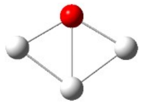

N.3.1.A ($^2A_1$, C_{2v})
0.00

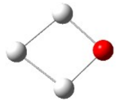

N.3.1.B ($^2A_1$, C_{2v})
0.06

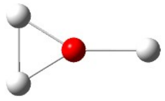

N.3.1.C ($^2A_1$, C_{2v})
0.38

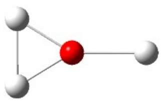

C.3.1.A ($^1A_1$, C_{2v})
0.00

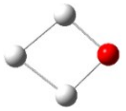

C.3.1.B ($^1A_1$, C_{2v})
0.32

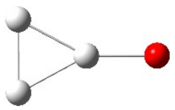

C.3.1.C ($^1A_1$, C_{2v})
1.35

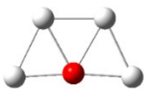

N.4.1.A ($^1A_1$, C_{2v})
0.00

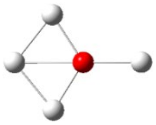

N.4.1.B ($^1A_1$, C_{2v})
0.16

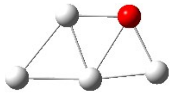

N.4.1.C ($^1A'$, C_s)
0.41

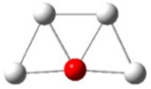

C.4.1.A ($^2B_2$, C_{2v})
0.00

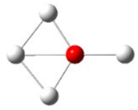

C.4.1.B ($^2A_1$, C_{2v})
0.23

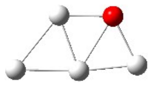

C.4.1.C ($^2A'$, C_s)
0.30

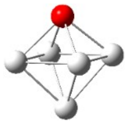

N.5.1.A ($^2B_1$, C_{4v})
0.00

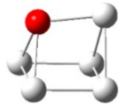

N.5.1.B ($^2A'$, C_s)
0.40

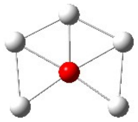

N.5.1.C ($^2B_2$, C_{2v})
1.04

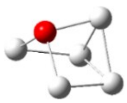

C.5.1.A ($^1A'$, C_s)
0.00

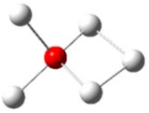

C.5.1.B ($^1A_1$, C_{2v})
0.35

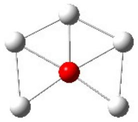

C.5.1.C ($^1A_1$, C_{2v})
0.39

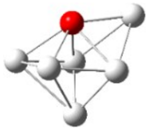

N.6.1.A ($^1A'$, C_s)
0.00

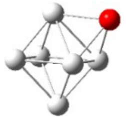

N.6.1.B ($^1A_1$, C_{3v})
0.08

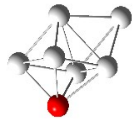

N.6.1.C ($^1A'$, C_s)
0.10

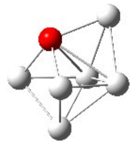

C.6.1.A (^{2}A’, C_s)
0.00

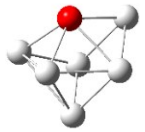

C.6.1.B (^{2}A, C_1)
0.03

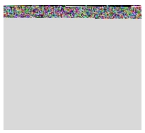

C.6.1.C (^{2}A’, C_s)
0.10

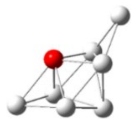

N.7.1.A (^{2}A’, C_s)
0.00

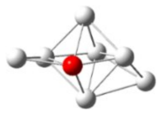

N.7.1.B (^{2}A, C_1)
0.13

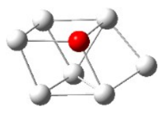

N.7.1.C (^{2}A, C_1)
0.22

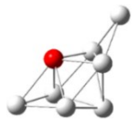

C.7.1.A (^{1}A’, C_s)
0.00

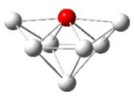

C.7.1.B (^{1}A$_1$, C_{2v})
0.01

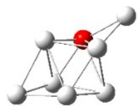

C.7.1.C (^{1}A, C_1)
0.15

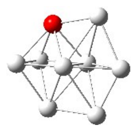

N.8.1.A (^{1}A’, C_s)
0.00

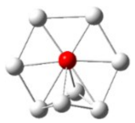

N.8.1.B (^{1}A’, C_s)
0.38

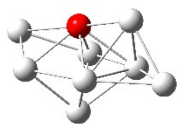

N.8.1.C (^{1}A’, C_s)
0.50

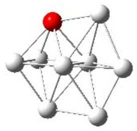

C.8.1.A (^{2}A’, C_s)
0.00

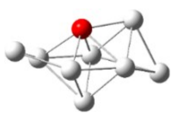

C.8.1.B (^{2}A’, C_s)
0.25

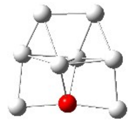

C.8.1.C (^{2}A, C_2)
0.34

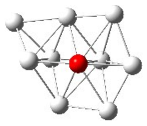

N.9.1.A (^{2}A, C_1)
0.00

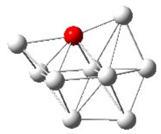

N.9.1.B (^{2}A’, C_s)
0.04

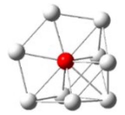

N.9.1.C (^{2}B$_2$, C_{2v})
0.40

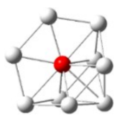

C.9.1.A (^{1}A$_1$, C_{2v})
0.00

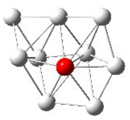

C.9.2.B (^{1}A, C_1)
0.17

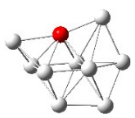

C.9.2.C (^{1}A’, C_s)
0.24

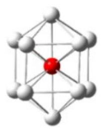

N.10.1.A (^{1}A, C_2)
0.00

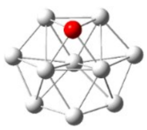

N.10.1.B (^{1}A’, C_s)
0.32

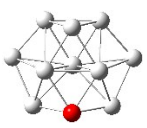

N.10.1.C (^{1}A’, C_s)
0.36

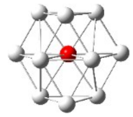

C.10.1.A ($^2A_1$, C_{2v})
0.00

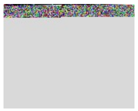

C.10.1.B (^2A, C_1)
0.11

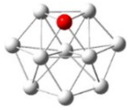

C.10.1.C (^2A, C_1)
0.16

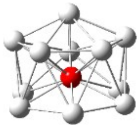

N.11.1.A ($^2A'$, C_s)
0.00

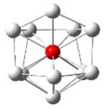

N.11.1.B ($^2A'$, C_s)
0.33

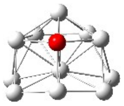

N.11.1.C ($^2A'$, C_s)
0.35

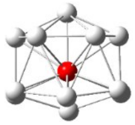

C.11.1.A ($^1A'$, C_s)
0.00

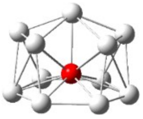

C.11.1.B ($^1A'$, C_s)
0.01

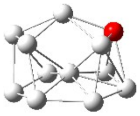

C.11.1.C ($^1A'$, C_s)
0.30

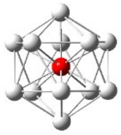

N.12.1.A (^1A_g, I_h)
0.00

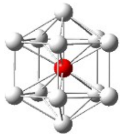

N.12.1.B ($^1A_1'$, D_{5h})
0.66

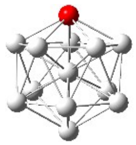

N.12.1.C ($^1A'$, C_s)
0.70

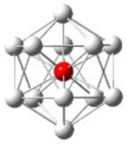

C.12.1.A ($^2A_{2u}$, D_{3d})
0.00

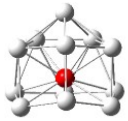

C.12.1.B ($^2A'$, C_s)
0.68

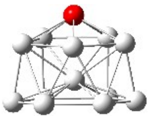

C.12.1.C ($^2A'$, C_s)
0.83

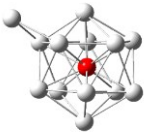

N.13.1.A ($^2B_1$, C_{2v})
0.00

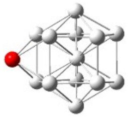

N.13.1.B ($^1A_1$, C_{2v})
0.62

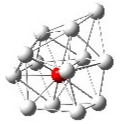

N.13.1.C ($^1A_1$, C_{2v})
0.66

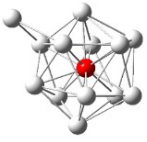

C.13.1.A ($^1A'$, C_s)
0.00

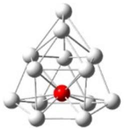

C.13.1.B ($^1A'$, C_s)
0.67

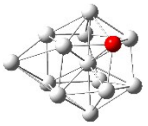

C.13.1.C (^1A, C_1)
0.71

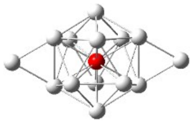

N.14.1.A (^1A_g, D_{2h})
0.00

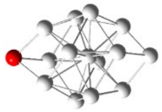

N.14.1.B ($^1A_1$, C_{2v})
0.03

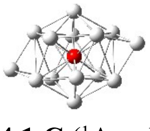

N.14.1.C ($^1A_{1g}$, D_{3d})
0.11

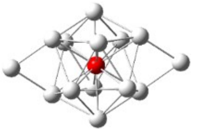

C.14.1.A (^{2}B$_u$, D_{2h})
0.00

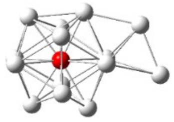

C.14.1.B (^{2}A", C_s)
0.28

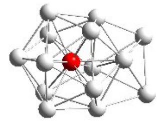

C.14.1.B (^{2}A, C_1)
0.35

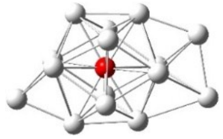

N.15.1.A (^{2}A', C_s)
0.00

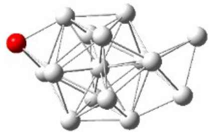

N.15.1.B (^{2}A', C_s)
0.21

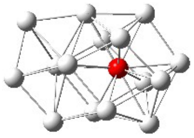

N.15.1.C (^{2}A', C_s)
0.46

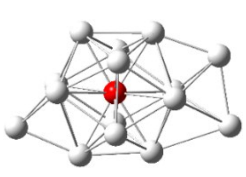

C.15.1.A (^{1}A', C_s)
0.00

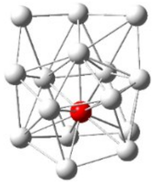

C.15.1.B (^{1}A, C_1)
0.41

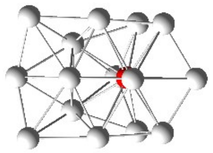

C.15.1.C (^{1}A', C_s)
0.43

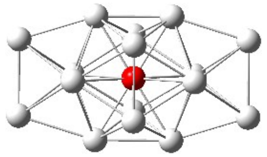

N.16.1.A (^{1}A$_g$, D_{2h})
0.00

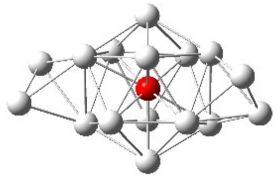

N.16.1.B (^{1}A, C_2)
0.24

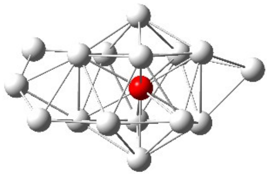

N.16.1.B (^{1}A, C_2)
0.34

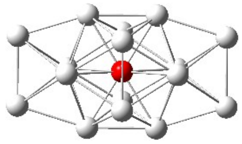

C.16.1.A (^{2}B$_{2u}$, D_{2h})
0.00

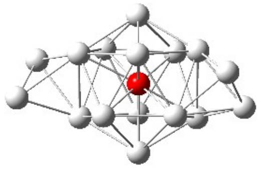

C.16.1.B (^{2}A", C_s)
0.46

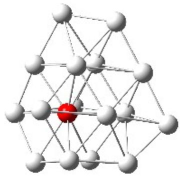

C.16.1.B (^{2}A, C_1)
0.58

Figure S1. Shapes, electronic states and relative energies (ΔE, eV) of the lower-lying isomers Al_nSi with n = 3 – 16 at the neutral and cationic states. ΔE values are obtained from (U)CCSD(T)/cc-pVTZ + ZPE computations.

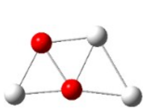

N.3.2.A (^{2}A', C_s)
0.00

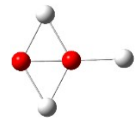

N.3.2.B (^{2}A'', C_s)
0.21

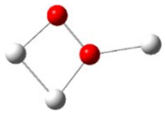

C.3.2.A (^{1}A, C_1)
0.00

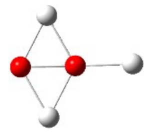

C.3.2.B (^{1}A$_1$, C_{2v})
0.07

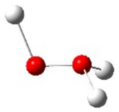

C.3.2.C (^{1}A', C_s)
0.76

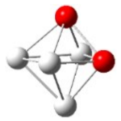

N.4.2.A (^{1}A, C_2)
0.00

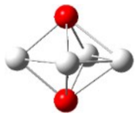

N.4.2.B (^{1}A$_1$, C_{2v})
0.35

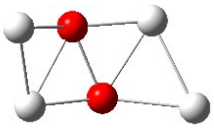

N.4.2.C (^{1}A, C_2)
1.34

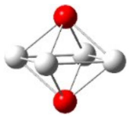

C.4.2.A (^{2}B$_{2g}$, D_{4h})
0.00

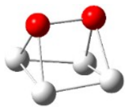

C.4.2.B (^{2}A, C_2)
0.23

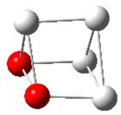

C.4.2.C (^{2}A', C_s)
0.34

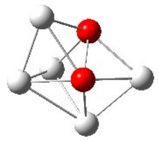

N.5.2.A (^{2}A, C_1)
0.00

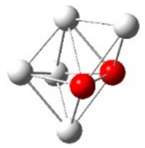

N.5.2.B (^{2}A', C_s)
0.03

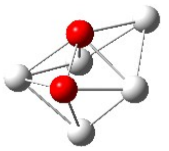

N.5.2.C (^{2}A, C_1)
0.10

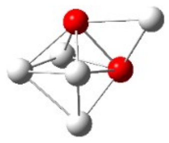

C.5.2.A (^{1}A, C_1)
0.00

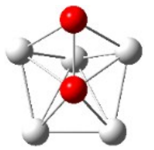

C.5.2.B (^{1}A', C_s)
0.14

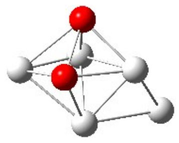

C.5.2.C (^{1}A, C_1)
0.23

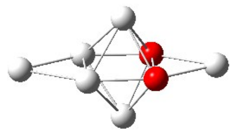

N.6.2.A (^{1}A$_1$, C_{2v})
0.00

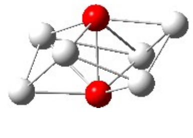

N.6.2.B (^{1}A, C_2)
0.06

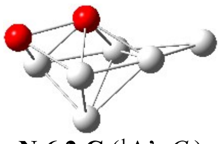

N.6.2.C (^{1}A', C_s)
0.26

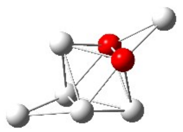

C.6.2.A (^{2}A', C_s)
0.00

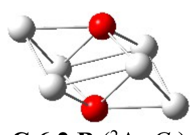

C.6.2.B (^{2}A, C_2)
0.08

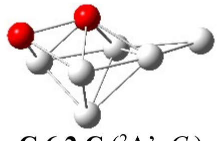

C.6.2.C (^{2}A', C_s)
0.12

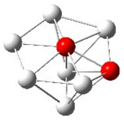

N.7.2.A (^{2}A, C_1)
0.00

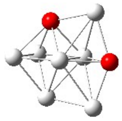

N.7.2.B (^{2}A’, C_s)
0.12

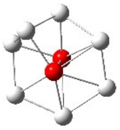

N.7.2.C (^{2}A, C_1)
0.20

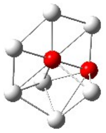

C.7.2.A (^{1}A, C_1)
0.00

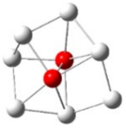

C.7.2.B (^{1}A, C_2)
0.08

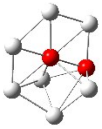

C.7.2.C (^{1}A, C_1)
0.24

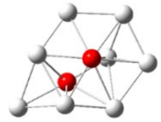

N.8.2.A (^{1}A, C_1)
0.00

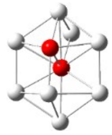

N.8.2.B (^{1}A’, C_s)
0.08

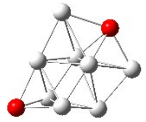

N.8.2.C (^{1}A’, C_s)
0.12

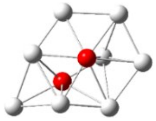

C.8.2.A (^{2}A, C_1)
0.00

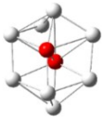

C.8.2.B (^{2}A, C_1)
0.18

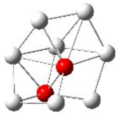

C.8.2.C (^{2}A, C_1)
0.26

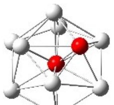

N.9.2.A (^{2}A", C_s)
0.00

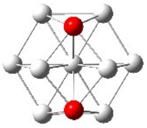

N.9.2.B (^{2}B$_1$, C_{2v})
0.15

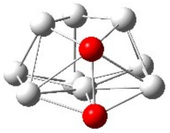

N.9.2.C (^{2}A’, C_s)
0.16

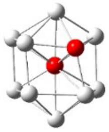

C.9.2.A (^{1}A, C_s)
0.00

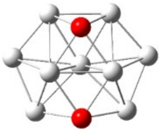

C.9.2.B (^{1}A’, C_s)
0.21

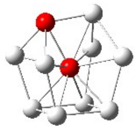

C.9.2.C (^{1}A’, C_s)
0.40

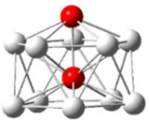

N.10.2.A (^{1}A’, C_s)
0.00

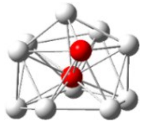

N.10.2.B (^{1}A, C_1)
0.01

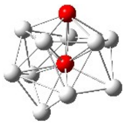

N.10.2.C (^{1}A, C_1)
0.11

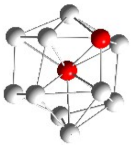

C.10.2.A (^{2}A, C_1)
0.00

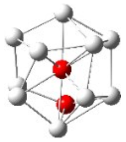

C.10.2.B (^{2}A, C_1)
0.05

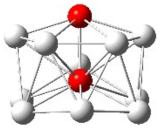

C.10.2.C (^{2}A’, C_s)
0.06

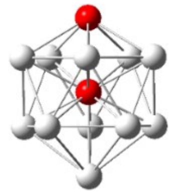

N.11.2.A ($^{2}A_1$, C_{3v})
0.00

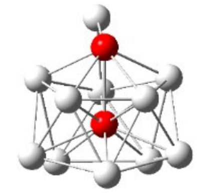

N.11.2.B (^{2}A, C_1)
0.78

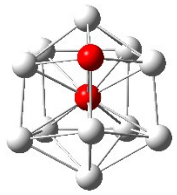

N.11.2.C ($^{2}A'$, C_s)
0.81

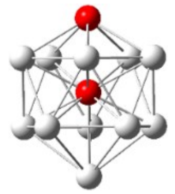

C.11.2.A ($^{1}A'$, C_s)
0.00

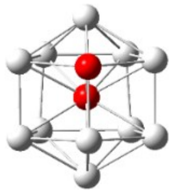

C.11.2.B ($^{1}A'$, C_s)
0.43

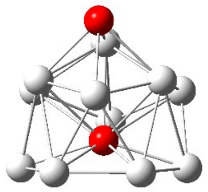

C.11.2.C ($^{1}A'$, C_s)
1.01

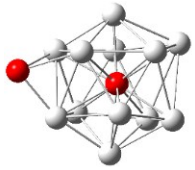

N.12.2.A ($^{1}A'$, C_s)
0.00

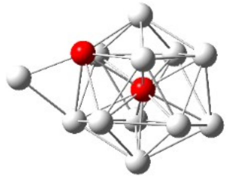

N.12.2.B ($^{1}A'$, C_s)
0.05

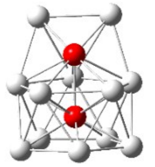

N.12.2.C (^{1}A, C_1)
0.16

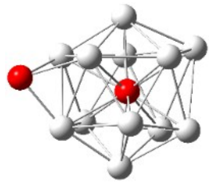

C.12.2.A ($^{2}A'$, C_s)
0.00

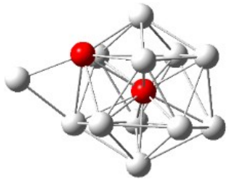

C.12.2.B ($^{2}A'$, C_s)
0.01

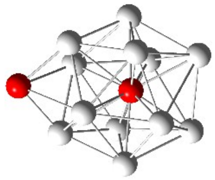

C.12.2.C (^{2}B, C_2)
0.08

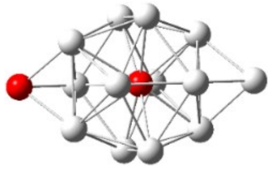

N.13.2.A ($^{2}A_1$, C_{3v})
0.00

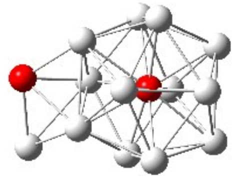

N.13.2.B ($^{2}A''$, C_s)
0.33

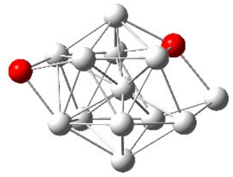

N.13.2.C ($^{2}A'$, C_s)
0.48

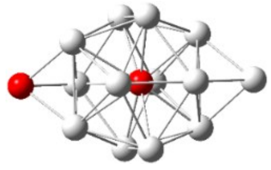

C.13.2.A ($^{1}A_1$, C_{3v})
0.00

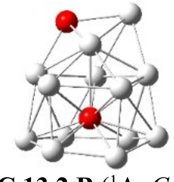

C.13.2.B (^{1}A, C_1)
0.36

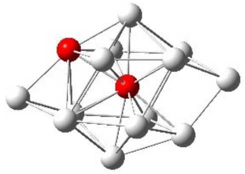

C.13.2.C ($^{1}A'$, C_s)
0.38

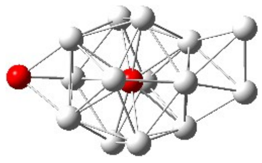

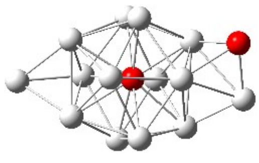

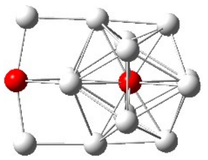

N.14.2.A (^{1}A’, C_s) 0.00 | **N.14.2.B** (^{1}A’, C_s) 0.23 | **N.14.2.C** (^{1}A, C_2) 0.27

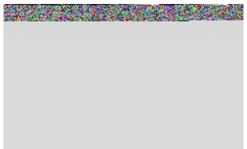
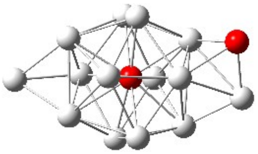
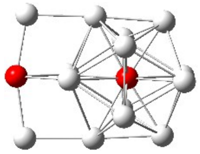

C.14.2.A (^{2}A’, C_s) 0.00 | **C.14.2.B** (^{2}A’, C_s) 0.22 | **C.14.2.C** (^{2}B$_1$, C_{2v}) 0.24

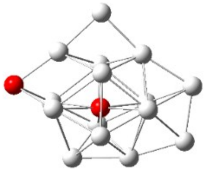
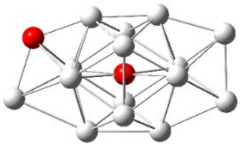
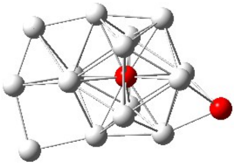

N.15.2.A (^{2}A’, C_s) 0.00 | **N.15.2.B** (^{2}A’, C_s) 0.02 | **N.15.2.C** (^{2}A, C_1) 0.11

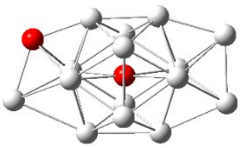
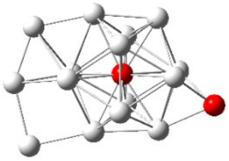
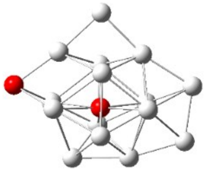

C.15.2.A (^{1}A’, C_s) 0.00 | **C.15.2.B** (^{1}A’, C_s) 0.04 | **C.15.2.C** (^{1}A, C_1) 0.46

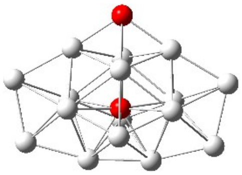
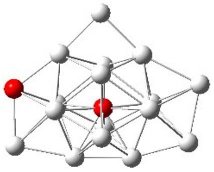
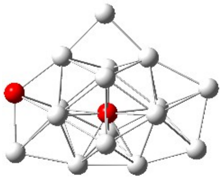

N.16.2.A (^{1}A$_1$, C_{2v}) 0.00 | **N.16.2.B** (^{1}A’, C_s) 0.01 | **N.16.2.C** (^{1}A’, C_s) 0.03

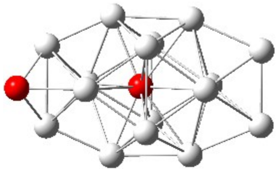
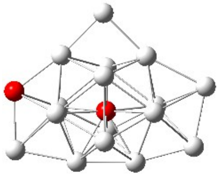
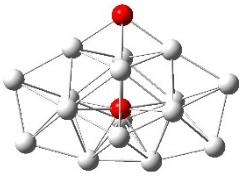

C.16.2.A (^{2}A”, C_s) 0.00 | **C.16.2.B** (^{2}A”, C_s) 0.07 | **C.16.2.C** (^{2}A, C_s) 0.09

Figure S2. Shapes, electronic states and relative energies (ΔE, eV) of the lower-lying isomers Al_nSi_2 with n = 3 – 16 at the neutral and cationic states. ΔE values are obtained from (U)CCSD(T)/cc-pVTZ + ZPE computations.

2. Details of electronic transitions corresponding to absorption peaks A and B of the most stable isomers in Figure 6.

Table S1. Absorption energy (eV), absorption wave length (nm), oscillator strength and major contributions corresponding to the absorption peaks A and B of the most stable isomers displayed in Figure 6.

Cluster	Peak	Absorption energy (eV)	Absorption wavelength (nm)	Oscillator strength, *f*	Major contributions	
					Initial MO	Final MO
Al_{13}^-	A	3.59	345	0.1339	HOMO-6 (2P)<br>HOMO-5 (2P)<br>HOMO-4 (2P)	LUMO+5 (3S)
	B	2.85	435	0.0991	HOMO-9 (1F)<br>HOMO-8 (1F)<br>HOMO-7 (1F)<br>HOMO-6 (2P)<br>HOMO-5 (2P)<br>HOMO-4 (2P)	LUMO (2D)<br>LUMO+1 (2D)<br>LUMO+2 (2D)<br>LUMO+3 (2D)<br>LUMO+4 (2D)
$Al_{12}Si$, isomer **N.12.1.A**	A	3.91	316	0.0391	HOMO-9 (2P)<br>HOMO-8 (2P)<br>HOMO-7 (2P)	LUMO+5 (3S)
	B	3.08	403	0.1064	HOMO-9 (2P)<br>HOMO-8 (2P)<br>HOMO-7 (2P)<br>HOMO-6 (1F)<br>HOMO-5 (1F)<br>HOMO-4 (1F)	LUMO (2D)<br>LUMO+1 (2D)<br>LUMO+2 (2D)<br>LUMO+3 (2D)<br>LUMO+4 (2D)
$Al_{11}Si^+$, isomer **C.11.2.A**	A	4.08	303	0.0169	HOMO-7 (1F)<br>HOMO-6 (2P)	LUMO+5 (3S)<br>LUMO+6 (1G)
	B	3.18	394	0.0778	HOMO-9 (2P)<br>HOMO-8 (1F)<br>HOMO-7 (2P)<br>HOMO-6 (2P)<br>HOMO-5 (1F)<br>HOMO-4 (1F)	LUMO (2D)<br>LUMO+1 (2D)<br>LUMO+2 (2D)<br>LUMO+3 (2D)<br>LUMO+4 (2D)

3. MO diagrams for the $Al_{12}Si^0$ cluster-isomer N.12.1.B and the $Al_{11}Si_2^+$ cluster-isomer C.11.2.B.

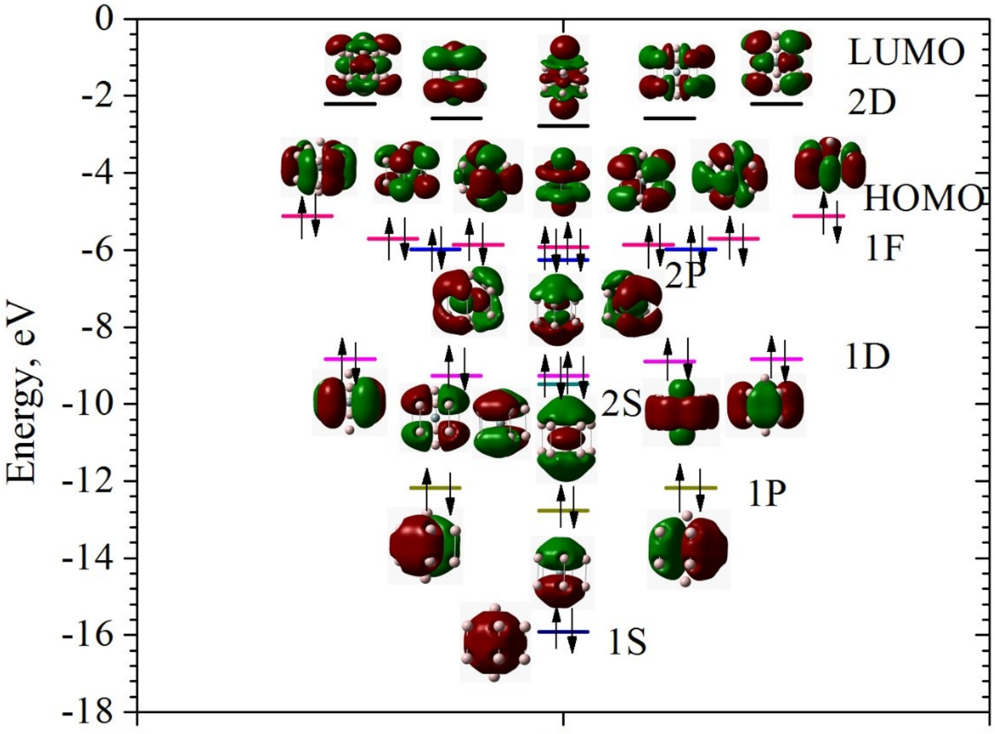

Figure S3. MO diagram of $Al_{12}Si$ neutral cluster, isomer N.12.1.B

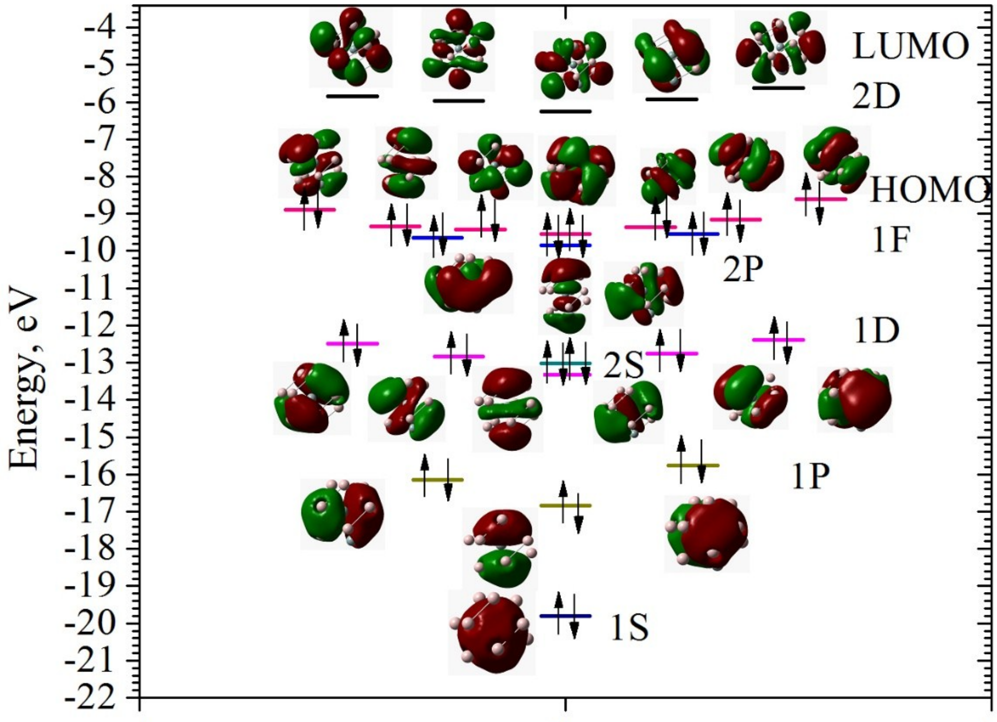

***Figure S4**. MO diagram of $Al_{11}Si_2^+$ cationic cluster, isomer C.11.2.B*

4. Occupation numbers and energies of the shell MOs of the investigated clusters

***Table S2**. Occupation numbers and energies of shell MOs of the Al_{13}^- anionic cluster (B3LYP/6-311+G(d))*

MO	Occupation number	Energy, a.u.	Energy, eV
91(LUMO+5, 3S shell MO)	0	0.06626	1.803027
90(LUMO+4, 2D shell MO)	0	0.01314	0.357558
89(LUMO+3, 2D shell MO)	0	0.01314	0.357558
88(LUMO+2, 2D shell MO)	0	0.01314	0.357558
87(LUMO+1, 2D shell MO)	0	0.01314	0.357558
86 (LUMO, 2D shell MO)	0	0.01314	0.357558
85 (HOMO, 1F shell MO)	2	-0.08227	-2.23868
84 (HOMO-1, 1F shell MO)	2	-0.08227	-2.23868
83 (HOMO-2, 1F shell MO)	2	-0.08227	-2.23868
82 (HOMO-3, 1F shell MO)	2	-0.08227	-2.23868
81 (HOMO-4, 1F shell MO)	2	-0.0864	-2.35106
80 (HOMO-5, 1F shell MO)	2	-0.0864	-2.35106
79 (HOMO-6, 1F shell MO)	2	-0.0864	-2.35106
78 (HOMO-7, 2P shell MO)	2	-0.09962	-2.7108
77 (HOMO-8, 2P shell MO)	2	-0.09962	-2.7108
76 (HOMO-9, 2P shell MO)	2	-0.09962	-2.7108
75 (HOMO-10, 2S shell MO)	2	-0.18167	-4.9435
74 (HOMO-11, 1D shell MO)	2	-0.21433	-5.83222
73 (HOMO-12, 1D shell MO)	2	-0.21433	-5.83222
72 (HOMO-13, 1D shell MO)	2	-0.21433	-5.83222
71 (HOMO-14, 1D shell MO)	2	-0.21433	-5.83222
70 (HOMO-15, 1D shell MO)	2	-0.21433	-5.83222
69 (HOMO-16, 1P shell MO)	2	-0.33289	-9.0584
68 (HOMO-17, 1P shell MO)	2	-0.33289	-9.0584
67 (HOMO-18, 1P shell MO)	2	-0.33289	-9.0584
66 (HOMO-19, 1S shell MO)	2	-0.4323	-11.7635

Table S3. *Occupation numbers and energies of shell MOs of the $Al_{12}Si$ neutral cluster: isomer* **N.12.1.A** *(B3LYP/6-311+G(d)).*

MO	Occupation number	Energy, a.u.	Energy, eV
91(LUMO+5, 3S shell MO)	0	-0.05785	-1.57418
90(LUMO+4, 2D shell MO)	0	-0.09911	-2.69692
89(LUMO+3, 2D shell MO)	0	-0.09911	-2.69692
88(LUMO+2, 2D shell MO)	0	-0.09912	-2.69719
87(LUMO+1, 2D shell MO)	0	-0.09912	-2.69719
86 (LUMO, 2D shell MO)	0	-0.09913	-2.69747
85 (HOMO, 1F shell, the higher MO)	2	-0.20074	-5.46242
84 (HOMO-1, 1F shell, the higher MO)	2	-0.20075	-5.46269
83 (HOMO-2, 1F shell, the higher MO)	2	-0.20079	-5.46378
82 (HOMO-3, 1F shell, the higher MO)	2	-0.20079	-5.46378
81 (HOMO-4, 2P shell MO)	2	-0.21401	-5.82351
80 (HOMO-5, 2P shell MO)	2	-0.21401	-5.82351
79 (HOMO-6, 2P shell MO)	2	-0.21402	-5.82378
78 (HOMO-7, 1F shell, the lower MO)	2	-0.22564	-6.13998
77 (HOMO-8, 1F shell, the lower MO)	2	-0.22566	-6.14052
76 (HOMO-9, 1F shell, the lower MO)	2	-0.22568	-6.14107
75 (HOMO-10, 1D shell MO)	2	-0.33439	-9.09922
74 (HOMO-11, 1D shell MO)	2	-0.33442	-9.10004
73 (HOMO-12, 1D shell MO)	2	-0.33444	-9.10058
72 (HOMO-13, 1D shell MO)	2	-0.33445	-9.10085
71 (HOMO-14, 1D shell MO)	2	-0.33449	-9.10194
70 (HOMO-15, 2S shell MO)	2	-0.34599	-9.41487
69 (HOMO-16, 1P shell MO)	2	-0.46564	-12.6707
68 (HOMO-17, 1P shell MO)	2	-0.46567	-12.6715

67 (HOMO-18, 1P shell MO)	2	-0.46571	-12.6726
66 (HOMO-19, 1S shell MO)	2	-0.60079	-16.3483

Table S4. *Occupation numbers and energies of shell MOs of the $Al_{11}Si_2^+$ cationic cluster – isomer* **C.11.2.A** *(B3LYP/6-311+G(d))*

**MO**	**Occupation number**	**Energy, a.u.**	**Energy, eV**
91(LUMO+5, 3S shell MO)	0	-0.17637	-4.79927
90(LUMO+4, 2D shell MO)	0	-0.22184	-6.03658
89(LUMO+3, 2D shell MO)	0	-0.2222	-6.04637
88(LUMO+2, 2D shell MO)	0	-0.22745	-6.18923
87(LUMO+1, 2D shell MO)	0	-0.22922	-6.2374
86 (LUMO, 2D shell MO)	0	-0.23086	-6.28202
85 (HOMO, 1F shell MO)	2	-0.33002	-8.98031
84 (HOMO-1, 1F shell MO)	2	-0.33074	-8.9999
83 (HOMO-2, 1F shell MO)	2	-0.33932	-9.23337
82 (HOMO-3, 1F shell MO)	2	-0.3405	-9.26548
81 (HOMO-4, 1F shell MO)	2	-0.34258	-9.32208
80 (HOMO-5, 1F shell MO)	2	-0.34302	-9.33405
79 (HOMO-6, the higher 2P shell MO)	2	-0.3524	-9.5893
78 (HOMO-7, the higher 2P shell MO)	2	-0.35275	-9.59882
77 (HOMO-8, 1F shell MO)	2	-0.3558	-9.68182
76 (HOMO-9, the lower 2P shell MO)	2	-0.36823	-10.0201
75 (HOMO-10, 1D shell MO)	2	-0.46476	-12.6468
74 (HOMO-11, 1D shell MO)	2	-0.46595	-12.6792
73 (HOMO-12, 1D shell MO)	2	-0.4705	-12.803

72 (HOMO-13, 1D shell MO)	2	-0.47141	-12.8277
71 (HOMO-14, 2S shell MO)	2	-0.47788	-13.0038
70 (HOMO-15, 1D shell MO whose shape mimics the 3d_z2 atomic orbital)	2	-0.495	-13.4696
69 (HOMO-16, 1P shell MO)	2	-0.59711	-16.2482
68 (HOMO-17, 1P shell MO)	2	-0.59735	-16.2547
67 (HOMO-18, 1P shell MO whose shape mimics the 2p_z atomic orbital)	2	-0.62942	-17.1274
66 (HOMO-19, 1S shell MO)	2	-0.744	-20.2453

5. A brief description of Jellium model

The jellium model has successfully been developed for the theoretical modelling of clusters for decades [1,2,3]. The jellium model considers a cluster of atoms as a superatom, where the cores of constituent atoms form a constant positive background, the so called "jellium density", and only valence electrons are treated explicitly. Under the model, the shell molecular orbitals of the clusters are denoted as 1S, 1P, 1D, 2S, 2P, 1F, … (usually the notation 1*s*, 1*p*, 1*d*, 2*s*, 2*p*, 1*f*, … have also been used), rather than as the atomic orbitals 1*s*, 2*s*, 2*p*, 3*s*, 3*p*, 3*d*, … of hydrogen-like atoms. [4]

6. A brief description of magic clusters

Magic clusters are clusters having all the energy levels are filled, or clusters having a certain number of atoms that are more stable than others. For instance, magic clusters are characterized by large ionization potentials that drop abruptly as one increase/decrease the cluster sizes by the addition/removal of an atom [5].

7. The shell MOs of the $Al_{11}Si_2^+$ cluster

For the cation $Al_{11}Si_2^+$, contributions of *s, p* and *d* AOs of all Al and two Si atoms into its shell MOs can be denoted as follows: 1S = 88% *s*(Si) + 4 %*p*(Si) + 8 %*s*(Al); 1P (for one lower 1P orbital) = 59% *p*(Si) + 15 %*s*(Si) + 2% *p*(Al) + 25 %*s*(Al); 1P (average value for two other higher 1P orbitals) = 55% *p*(Si) + 3% *p*(Al) + 41% *s*(Al); 2S = 1% *p*(Si) + 94%*s*(Si) + 0.4% *p*(Al) + 4% *s*(Al); 1D (for one lower 1D orbital) = 34% *p*(Si) + 39% *s*(Si) + 3% *p*(Al) + 23% *s*(Al); 1D (average value for two next higher 1D orbitals) = 47% *p*(Si) + 2% *s*(Si) + 4% *p*(Al) + 48% *s*(Al); 1D (average value for the two highest 1D orbitals) = 19%*p*(Si) + 7% *p*(Al) + 74% *s*(Al); 2P (average value for three 2P orbitals) = 53% *p*(Si) + 3% *s*(Si) + 4% *p*(Al) + 40% *s*(Al); 1F (average value for seven 1F orbitals) = 53% *p*(Si) + 3% *s*(Si) + 12% *p*(Al) + 47% *s*(Al).

Substitution of one of Al atoms on the surface of an icosahedron by the second Si atom not only induces a stronger attraction with electron along the Si-Si axis, but also lowering the symmetry of the $Al_{11}Si_2^+$ cluster as compared to the two other clusters. This constitutes a reason for not only the splitting of the 1F levels, but also for the 1P, 1D and 2P levels. Concretely, the P shell orbitals (1P and 2P) break into two different sub-levels, namely, the P shell along the Si-Si line is located at lower energy, and the two other P shell orbitals at higher position levels.

If we now set the Si-Si axis to be the z-axis, then we could see that the 1D shell orbitals split into three levels, namely the 1D whose shape is similar to the $3d_z2$ atomic orbital located even lower in energy than the 2S. The two other 1D levels whose shapes are similar to those of the 3d(xz) and 3d(yz) orbitals come next, and then the two other 1D whose shapes can be compared to those of the $3d_{(x}2_{-y}2_{)}$ and $3d_{xy}$ AOs. This is represented in Figure 5c of the MO diagram of the $Al_{11}Si_2^+$ cluster.

The splitting of 1F shell orbitals could be described as follows: those that are composed mainly of *s*(Al) AOs and those having lobes along the Si-Si axis, are energetically located below, and those having lobes farther away from the axes are located above.

8. Current density for Al_{13}^{-}, $Al_{12}Si$, and $Al_{11}Si_2^{+}$ at several parallel planes of located above and below the center atoms

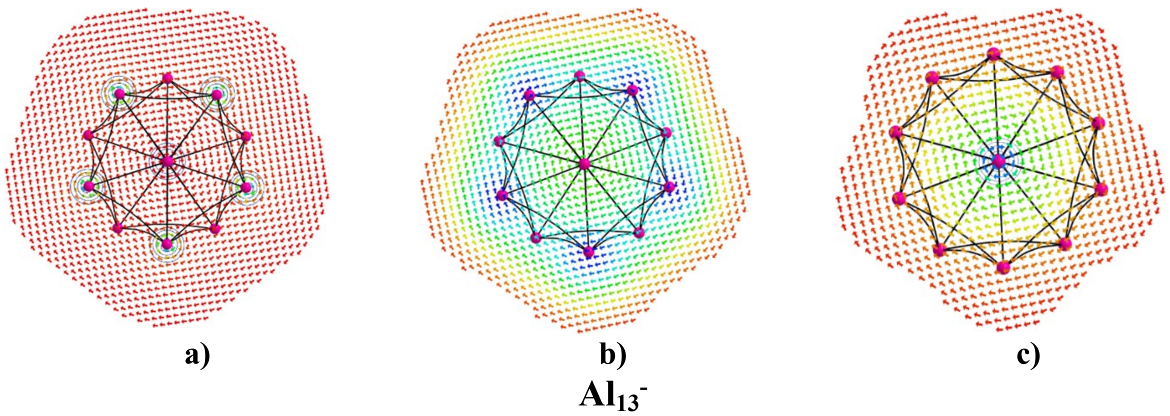

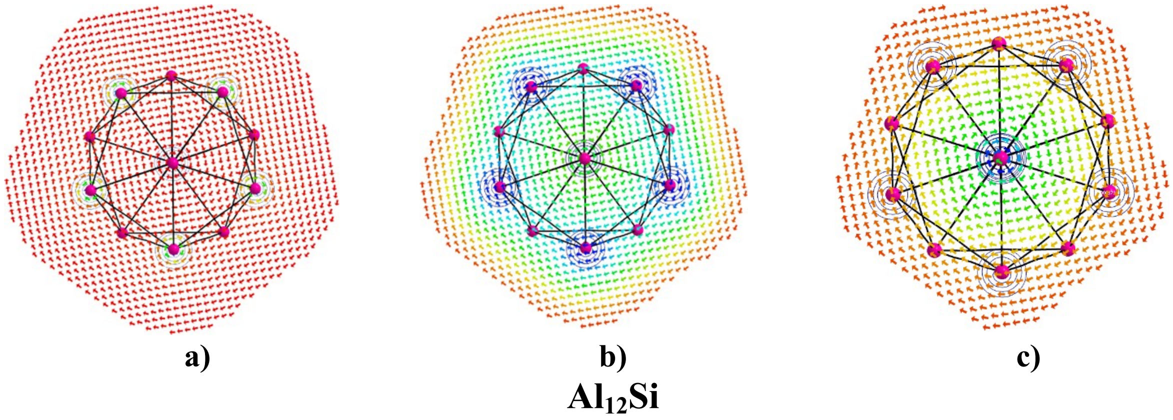

Figure S5. Profiles of current density for Al_{13}^{-} and $Al_{12}Si$ at the planes located above and below the center atoms by **a)** ± 2 Bohr; **b)** ± 4 Bohr; and **c)** ± 6 Bohr. Red to blue arrows represents weak to strong current density with the range: 0 to 0.0006 a.u.

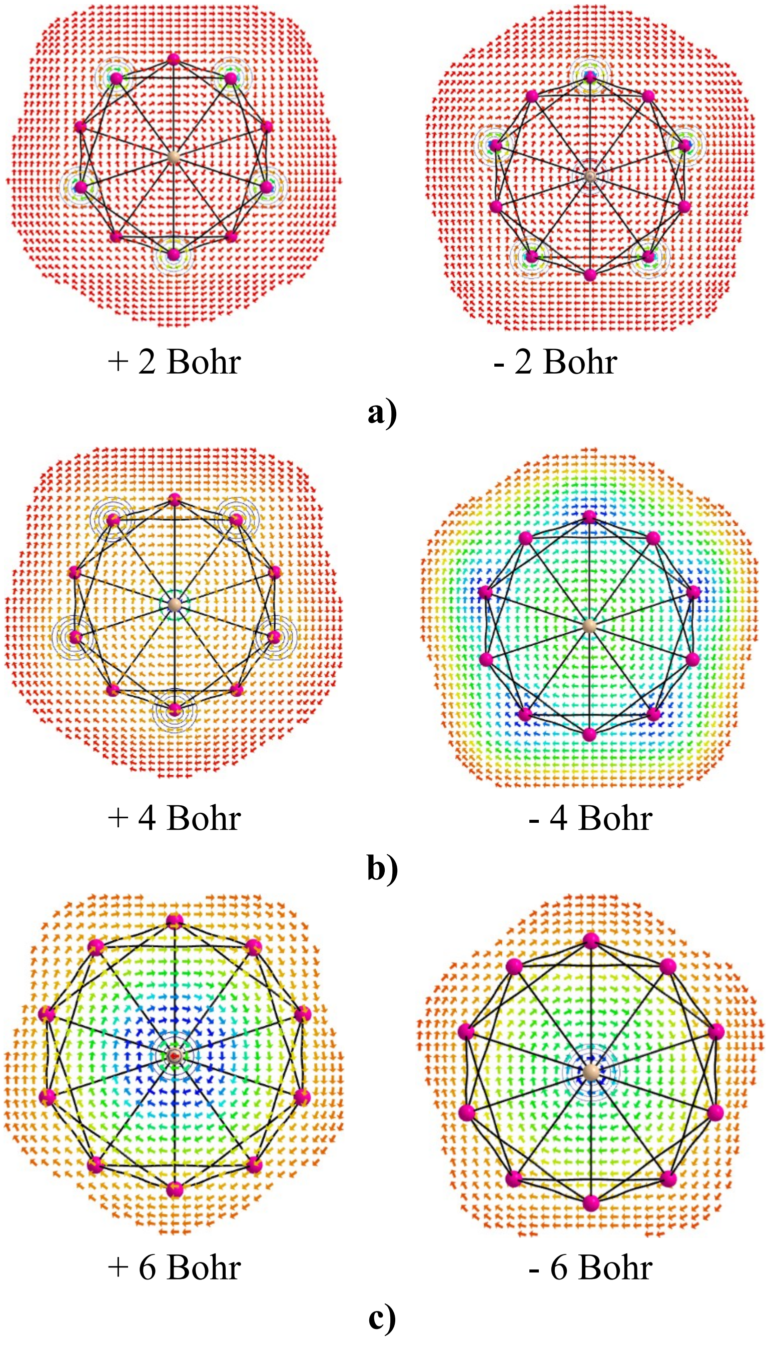

Figure S5. Profiles of current density for $Al_{11}Si_2^+$ at the planes located above below the center atoms by **a)** ± 2 Bohr; **b)** ± 4 Bohr; and **c)** ± 6 Bohr. Red to blue arrows represents weak to strong current density with the range: 0 to 0.0006 a.u.

References

[1] Zhenyang Lin, Tom Slee, D.M.P.Mingos, "A structural jellium model of cluster electronic structure", Chemical Physics, Volume **142** (1990), pages 321-334.

[2] M. Koskinen, P.O. Lipas, M. Manninen, "Electron-gas clusters: the ultimate jellium model", z. Phys. D, Volume **35** (1995) pages 285-297.

[3] N. D. Lang and W. Kohn, "Theory of Metal Surfaces: Charge Density and Surface Energy", Phys. Rev. B., Vol. 1, 1970, pages 4555-4568.

[4]. Günter Schmid (Ed.), Clusters and Colloids: From Theory to Applications, VCH Publishers, Inc., New York, NY (USA), 1994.

[5] Julius Jellinek (Ed.), Theory of Atomic and Molecular Clusters With a Glimpse at Experiments, Springer, 1999, pages 27-31.
